# Importance of Primary and Secondary Hydrogen Bonding Interactions of Polyols on the Plasticization of Chitosan

**DOI:** 10.1021/acsomega.4c05696

**Published:** 2024-09-24

**Authors:** Seth W. Mars, Brandy L. Davidson, Kayla H. Moore, Brycelyn M. Boardman, Gretchen M. Peters

**Affiliations:** Department of Chemistry and Biochemistry, James Madison University, Harrisonburg, Virginia 22807, United States

## Abstract

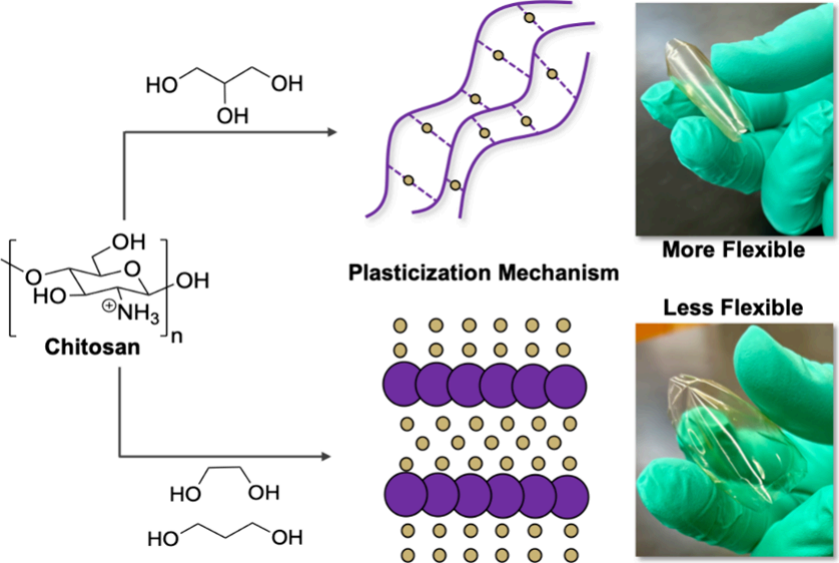

Molecular-level interactions between glucosamine and glycerol have shed light on the specific binding motif required for the plasticization of chitosan with glycerol via the gel theory mechanism. Here, we describe a spectroscopic study of the intermolecular interactions between the monomeric repeat unit of chitosan, glucosamine, and simplified 1,2- and 1,3-diol units of glycerol (i.e, 1,3-propanediol and ethylene glycol). The material properties of chitosan films containing these diols at varying concentrations were characterized using ATR-IR, DMA, TGA, and SEM. The combined results indicate that these diols plasticize chitosan via the lubricity theory mechanism, which differs from glycerol that plasticizes via the gel theory mechanism. At low concentrations, this difference in mechanism has a minimal impact on the material properties. However, at high concentrations of the diols, the necessity of a secondary hydrogen bonding interaction for the retention of chitosan plasticization is observed with a significant increase in the Young’s modulus of the materials. The impact of hydrophobicity within the diols was also investigated in chitosan films using 1,2-propanediol and 2-methyl-1,3-propanediol. The combined analyses provide strong evidence that both primary and secondary interactions are responsible for determining the mechanism of chitosan plasticization.

## Introduction

Chitosan, a commercially available biopolymer derived from shrimp shells, is a nontoxic and inexpensive alternative to petroleum-based polyolefins. Its biocompatible nature and antibacterial properties^1^ make it an exciting material for a wide range of applications from food packaging^2^ and sensing to pharmaceutical^3,4^ and orthopedic applications.^5−7^ Recent reviews have also highlighted the versatility of chitosan hydrogels for their unique molecular-level structure and for their response to external stimuli, making them promising materials for diverse areas of research.^8,9^ Additionally, chitosan has also shown promise as an adsorbent material to remove a range of challenging pollutants in wastewater from fluoride ions^10^ to pharmaceutical contaminants.^11^ However, chitosan has not replaced its petroleum-based counterparts because in its natural state, chitosan is rigid and brittle—a function of the strong intermolecular hydrogen bonding between polymer chains. The investigation of plasticizers to improve the mechanical properties of chitosan by disrupting the intermolecular chain interactions of the polymer has been widely explored for a variety of applications.^12−20^ Polyols such as glycerol (Glyc) have the ability to improve flexibility in chitosan films by interacting with specific functionalities on the polymer backbone and disrupting the hydrogen bonding network of the polymer. This would suggest that increasing the hydrogen bonding capability of the plasticizer would result in improved materials. However, it has been shown that additives with strong hydrogen bonding capabilities, such as ionic liquids, are not invariably able to plasticize chitosan.^21^

**Figure 1:**
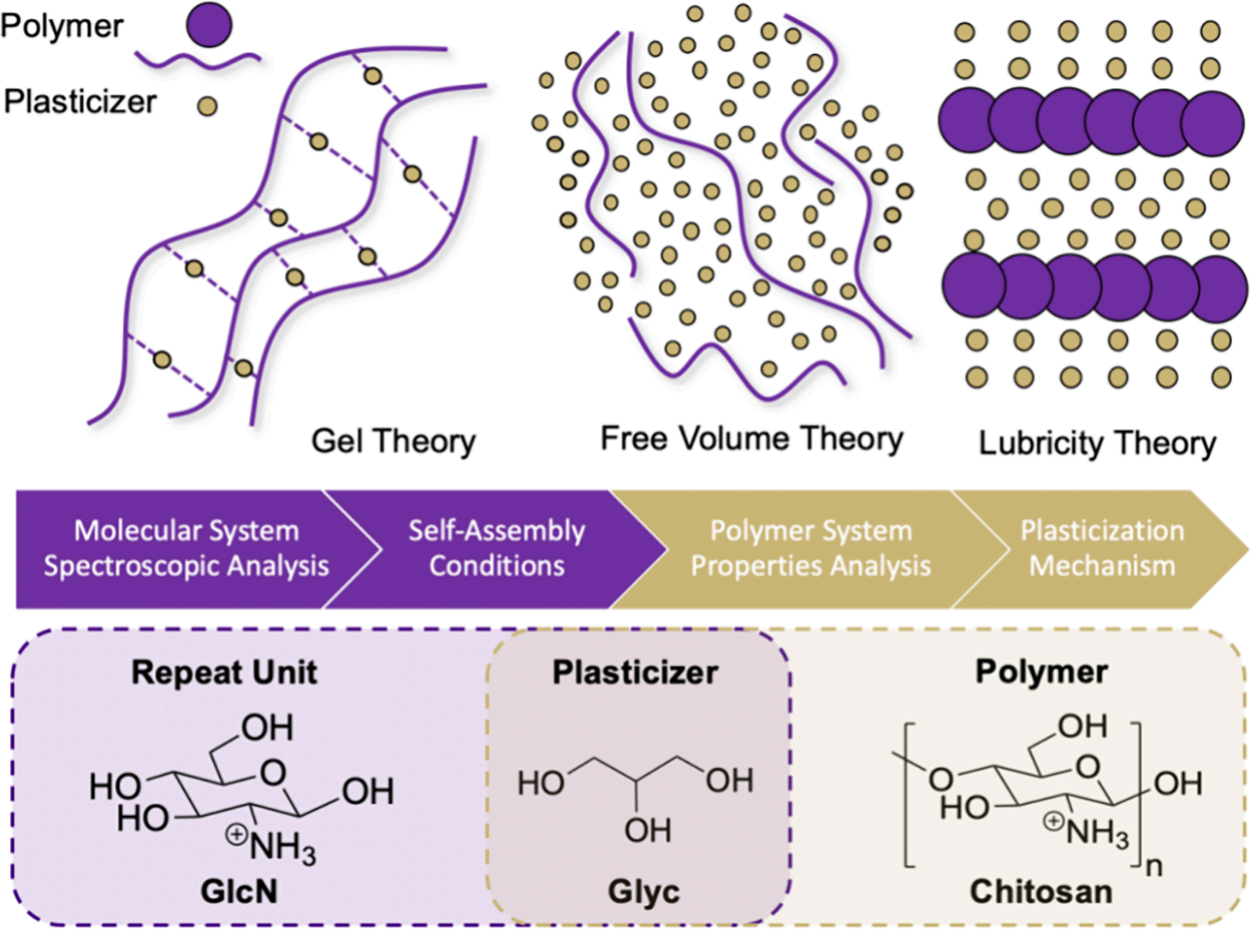
(Top) Schematic representation of plasticization theories. (Middle) Workflow of the process used to elucidate the plasticization theory present in chitosan systems using a combination of molecular-level analysis and polymer property characterization. (Bottom) Chemical structures of the molecules and polymer used in these processes. The structure of chitosan is shown as the protonated ammonium ion to reflect its state when exposed in acetic acid in the films.

It was postulated that to achieve effective plasticization of chitosan, the plasticizer needs to have hydrogen bonding capabilities to disrupt the interchain hydrogen bonding network but be incapable of forming cross-links between the chitosan chains. It is suggested that Glyc has this ideal capability where the OH groups hydrogen bond to chitosan and the hydrophobic C–H groups of the Glyc backbone inhibit hydrogen bonding cross-links. DFT calculations were performed using an *N*-acetylglucosamine derivative as a chitosan model in an effort to understand the interactions between chitosan and Glyc.^22^ The result of this work indicated that all three OHs of Glyc bind to *N*-acetylglucosamine and thus supported the chitosan plasticization model for Glyc via hydrogen bonding and hydrophobicity. This type of interaction would suggest that the plasticization of chitosan with Glyc would fall under the lubricity theory in which Glyc lubricates the movement between chains and forms alternating layers of plasticizer and polymer (Figure 1).^23^ However, the use of *N*-acetylglucosamine as a model for Glyc binding is problematic, because the degree of deacetylation has been shown to impact plasticization of the polymer.^21,24^ In most cases, 75–85% deacetylated chitosan is used, which would remove the three-coordinate binding ability of Glyc in the absence of the carbonyl functionality. In this case, the hydrophobic backbone of Glyc would not be exposed.

Conversely, reports also suggest that Glyc and other oils lead to the formation of new or increased numbers of existing bonds within the polymer structure. This increase affects the water affinity of the film and as a result changes the properties of the material.^25^ This type of interaction would correspond with the gel theory of plasticization where weak secondary interactions between the plasticizer and the polymer chains inhibit chain–chain interactions from reforming.^23^ These different reports highlight the poorly defined plasticization mechanism for Glyc and chitosan. In turn, this lack of knowledge inhibits the development of new plasticizers for chitosan and other relevant biopolymers. Our recent work investigated the interactions of glucosamine (GlcN), the repeat unit of chitosan (deacetylated), and Glyc in an attempt to elucidate the plasticization mechanism (Figure 1). Free volume theory was ruled out because specific hydrogen bonding motifs were observed between the NH of GlcN and Glyc while other GlcN-Glyc interactions were less defined. Similarly, lubricity theory was also less likely because GlcN–GlcN interactions were always observed, suggesting that the plasticizer cannot completely isolate GlcN molecules, even at high concentrations. Well-defined hydrogen bonding motifs and the persistence of GlcN–GlcN interactions would therefore suggest that gel theory is the best model for the plasticization of chitosan.^26^

**Figure 2:**
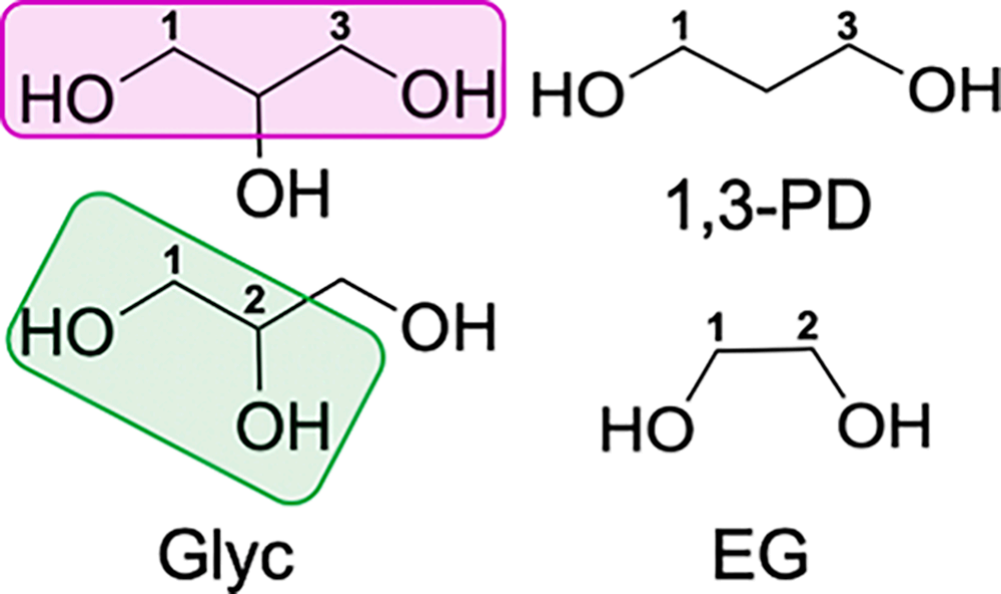
Structures of Glyc, 1,3-PD, and EG. Primary binding modes of Glyc are numbered and highlighted as 1,3 binding (fuschia) and 1,2 binding (green). Corresponding binding modes in 1,3-PD and EG are numbered.

Our previous work emphasized the importance of the binding face on the intermolecular interactions between the polyol and GlcN, as well as GlcN–GlcN self-assembly. Herein, we considered the impact of the polyol structure on these interactions and on chitosan plasticization. Simplifying the polyol structure into individual diol units allows us to categorize the interactions needed for different binding events and provides insights into the structural necessities of the plasticizer. That is, for Glyc, evaluating ethylene glycol (EG) and 1,3-propanediol (1,3-PD) isolates and differentiates the interactions of the 1,2-diol unit and 1,3-diol unit, both of which are present in Glyc (Figure 2). Additionally, to probe secondary interactions and the importance of hydrophobicity versus hydrogen bonding, 1,2 propanediol (1,2-PD) and 2-methyl-1,3 propanediol (2-Me-1,3-PD) were also investigated. Molecular-level (GlcN) and polymeric (chitosan) systems were studied to build a strong fundamental understanding of the specific structural and hydrogen bonding requirements for effective plasticization. This knowledge has been previously lacking and has the potential to impact the future design of more efficient plasticizers in biopolymers.

## Results and Discussion

**Figure 3:**
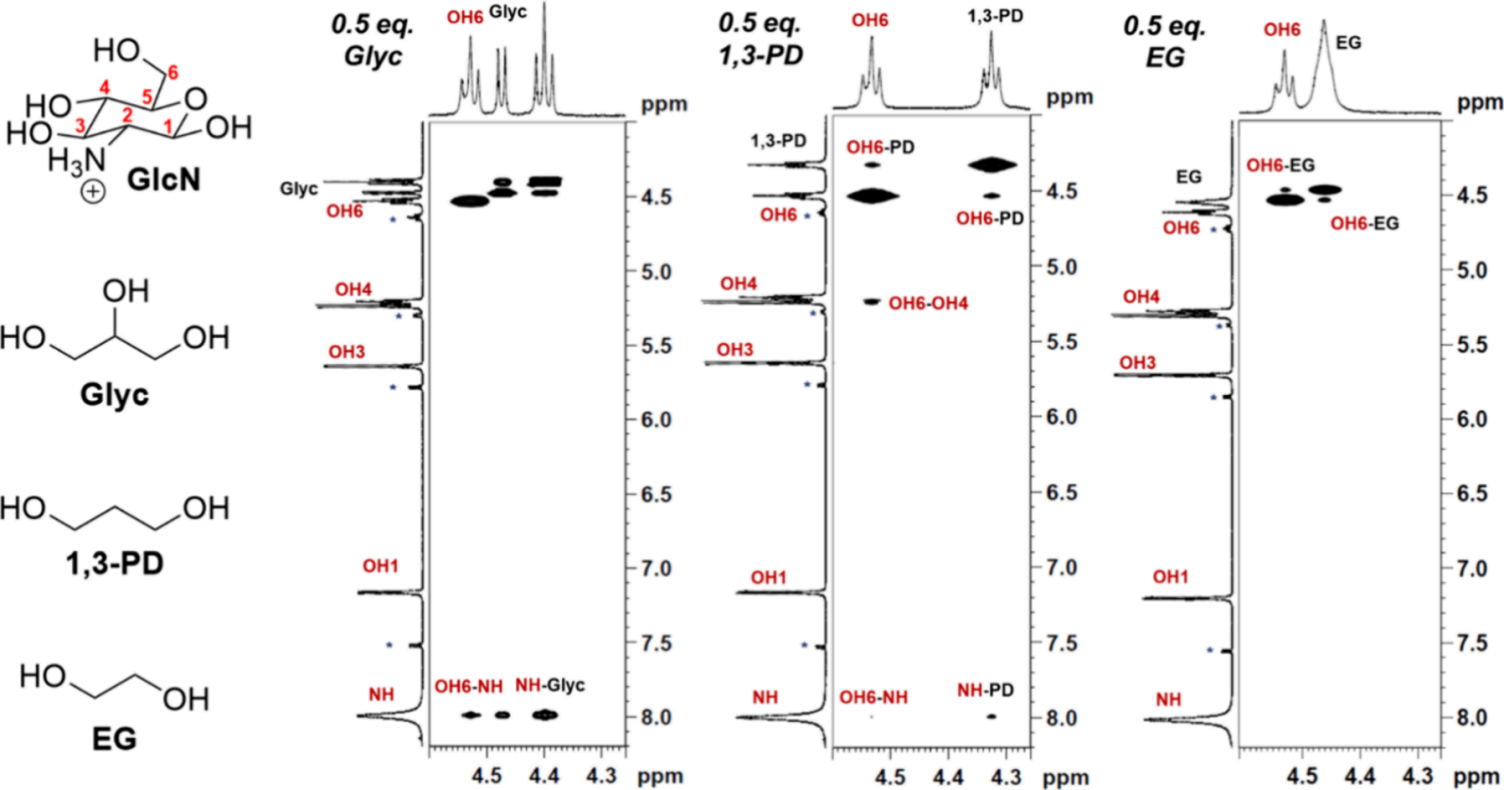
Representative cross sections of the 2D NOESY spectra of the polyol OHs at 12.5 mM GlcN and 6.25 mM Glyc (left), 6.25 mM 1,3-PD (middle), or 6.25 mM EG (right) recorded at 400 MHz (9.4 T). While Glyc strongly prefers to bind to the NH of GlcN (NH-Glyc), 1,3-PD has cross-peaks with OH6 (OH6-PD) and NH (NH-PD) and EG preferentially interacts with the OH6 (OH6-EG). Small peaks for the alpha anomer of GlcN are observed in the spectra, and the corresponding peaks are denoted with an asterisk (*).

**Figure 4:**
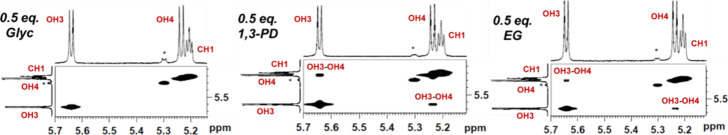
Representative cross sections of the 2D NOESY spectra of the OH3 and OH4 of GlcN at 12.5 mM GlcN and 6.25 mM Glyc (left), 6.25 mM 1,3-PD (middle), or 6.25 mM EG (right) recorded at 400 MHz (9.4 T). The presence of an OH3-OH4 cross-peak indicates that the intramolecular hydrogen bonding is still intact. Small peaks for the alpha anomer of GlcN are observed in the spectra, and the corresponding peaks are denoted with an asterisk (*).

**Figure 5:**
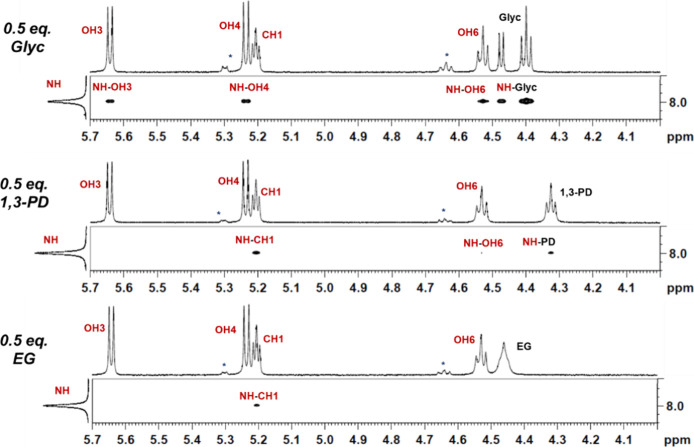
Representative cross sections of the 2D NOESY spectra of the NH of GlcN at 12.5 mM GlcN and 6.25 mM Glyc (top), 6.25 mM 1,3-PD (middle), or 6.25 mM EG (bottom) recorded at 400 MHz (9.4 T). The presence of NH–OH cross-peaks is indicative of GlcN–GlcN aggregation. Small peaks for the alpha anomer of GlcN are observed in the spectra, and the corresponding peaks are denoted with an asterisk (*).

To understand the impact of primary diol binding mode on plasticization, we first sought to evaluate the molecular-level interactions between our model compound, GlcN, and either EG or 1,3-PD. Nuclear Overhauser effect spectroscopy (NOESY) is a powerful tool for probing these interactions as it can elucidate any through space and chemical exchange (EXSY) interactions between protons that are in close proximity. In our previous work with Glyc, we found that Glyc had a strong and immediate propensity for the NH functionality of GlcN and that the Glyc was capable of disrupting intramolecular interactions while promoting GlcN–GlcN aggregation.^26^ Shown in Figures 3–5 are selected cross sections of the 2D NOESY spectra at 12.5 mM GlcN in *d*_6_-DMSO with 0.5 equiv of polyol. The full spectra and additional concentrations of polyol can be found in Figures S1–S10. A GlcN concentration of 12.5 mM was chosen as it corresponds with GlcN in a relatively “isolated” state and thus allows us to probe both the intermolecular GlcN–diol interactions and polyol-promoted GlcN–GlcN association. Notably, we observed signals for the alpha anomer of GlcN in all NMR spectra recorded. Signals for this anomer are indicated with asterisks (*) in each spectrum.

To start, we investigated the impact of diol binding mode on the intermolecular interactions between GlcN and the OHs of the polyol. As anticipated, with 0.5 equiv of Glyc, only one strong, positive cross-peak is observed between the OHs of Glyc and the NH of GlcN (Figure 3, NH-Glyc), indicating that there is chemical exchange and hydrogen bonding between these two protons. With 1,3-PD, we similarly see a positive cross-peak for this OH/NH interaction (NH-PD), but it is significantly less intense. Additionally, a prominent positive cross-peak between OH6 of GlcN and OH of the 1,3-PD is observed (OH6-PD). The relative intensity of these two NOE signals varies as the concentration of 1,3-PD is increased. At 0.5 equiv of 1,3-PD, the interaction between OH6-PD is more intense than the NH-PD cross-peak. However, as the concentration of 1,3-PD increases, the intensity of the NH-PD cross-peak increases, and the diol’s hydrogen bonding preference shifts to the amine of GlcN (Figures S1 and S2). Interestingly, with 0.5 equiv of EG, the only significant signal is a positive cross-peak between the OH6 of GlcN and the OH’s of EG (OH6-EG). This signal remains the predominant cross-peak at both 0.5 and 1.0 equiv of diol, and EG has no NH interactions until it is in excess (Figures S1 and S2). In combination, these data suggest that the 1,3-PD has a higher preference for the NH than EG, which has a higher preference for the OHs of GlcN. These results clearly demonstrate that the diol binding mode impacts the intermolecular interactions between GlcN and the polyol.

With insights into the intermolecular interactions between the polyol and GlcN, we next turned our attention to the intra- and intermolecular interactions of GlcN. Our previous work showed that Glyc was capable of disrupting intramolecular GlcN hydrogen bonds while promoting intermolecular GlcN–GlcN aggregation.^26^ We determined that the positive cross-peaks between OH3-OH4, OH3-OH6, and OH4-OH6 of GlcN were good diagnostic signals for intramolecular hydrogen bonding. Figure 4 shows the NOESY cross sections of GlcN with 0.5 equiv of the various polyols from 5.1 to 5.7 ppm. This portion of the spectrum includes signals for the OH3, OH4, and CH1 of GlcN and is therefore a useful region for monitoring the OH3-OH4 cross-peak and evaluating intramolecular GlcN hydrogen bonding. Interestingly, while this OH3-OH4 cross-peak indeed disappears in the presence of even substoichiometric amounts of Glyc, with either EG or 1,3-PD, it is maintained until excess diol is present (Figures S1 and S2). Additionally, other key intramolecular interactions, namely, the OH6-OH4 interactions, are still present when 1,3-PD is added to GlcN, but not with EG or Glyc (Figure 3, Figures S1 and S2). This suggests that neither EG nor 1,3-PD is as effective as Glyc at disrupting intramolecular GlcN interactions.

Lastly, to evaluate the propensity of the diols to promote intermolecular interactions between GlcN molecules, the GlcN–GlcN NH–OH interactions were evaluated. In our previous work, we found that when GlcN is isolated, the NH “sees” only the protons of its carbon neighbor (CH1). When aggregated, however, this NH–CH1 interaction is lost and a series of new NH–OH cross-peaks are observed.^26^ This is consistent with GlcN–GlcN self-association and aggregation. As expected, when 0.5 equiv of Glyc is added to a 12.5 mM GlcN solution in *d*_6_-DMSO, the NH–CH1 cross-peak disappears, and new cross-peaks for NH–OH3, NH–OH4, and NH–OH6 are observed (Figure 5). In contrast, when 0.5 equiv of 1,3-PD or EG is introduced to the system, the NH–CH1 NOE is maintained and little to no interactions are seen between the NH of GlcN and its OHs. Even at excess diol, no GlcN–GlcN self-assembly is observed (Figures S7 and S10). We interpret these results to mean that although EG and 1,3-PD readily bind to GlcN, they are not capable of promoting GlcN–GlcN aggregation.

**Figure 6:**
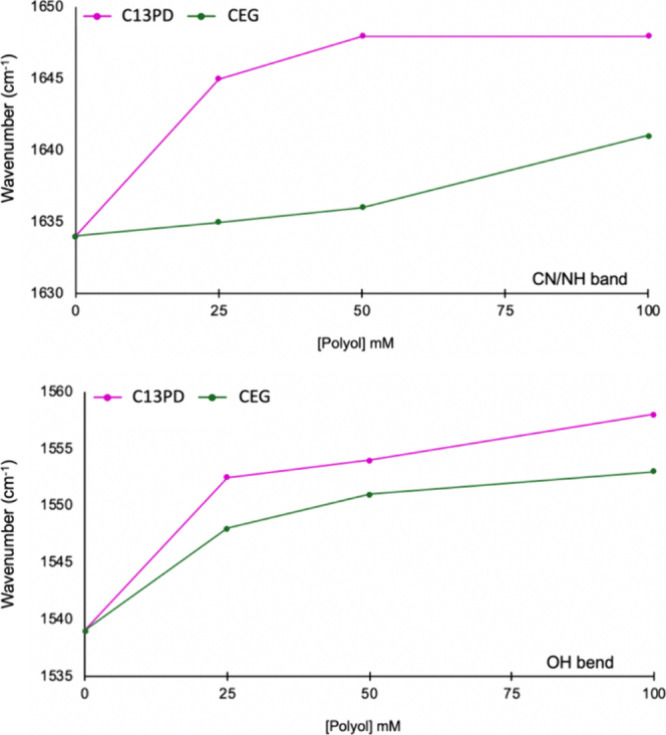
Change in wavenumber (cm^–1^) of the ATR-IR CN/NH band (top) and OH bend (bottom) with increasing polyol concentration for C13PD25-100 (fuschia) and CEG-25-100 (green).

We have previously demonstrated the correlation between molecular interactions and the resulting impacts on chitosan film properties with Glyc.^26^ For this study, films containing 1% chitosan (C) and 25.0 50.0 or 100.0 mM polyol (Glyc (CG-25-100), 13PD (C13PD-25-100), and EG (CEG-25-100)) were investigated. Pure chitosan films (PCF) and CG-25-100 were used as controls to evaluate the impacts of 1,3-PD and EG on the material properties. Films were initially analyzed by using attenuated total reflectance infrared spectroscopy (ATR-IR) to probe structural interactions. The frequency range from ∼1680 to 1500 cm^–1^ has been used diagnostically in the study of chitosan and Glyc interactions as it includes the C–N stretch, CN-H bend, and CO-H bend. Shifts to higher wavenumbers in the ATR-IR spectra indicate an increase in hydrogen bonding and are therefore useful tools in understanding structural interactions between the polymer and plasticizer molecules. At low concentrations of diol (25 mM), a shift in the CN/NH combination band from 1634 cm^–1^ in PCF to 1645 cm^–1^ for C13PD-25 and 1635 cm^–1^ for CEG-25 is observed. These results are in agreement with the molecular-level NMR findings, confirming that 1,3-PD has a preferred binding with NH when compared to EG in the polymeric system. Similar to the NMR, as the concentration of EG is increased, interactions with the NH are observed with an increasing shift in wavenumber of the CN/NH band (Figure 6, Figure S11). A consistent increase in wavenumber of the OH bend is observed for both C13PD and CEG with increasing concentration of polyol. At all concentrations of 1,3-PD, the shift in wavenumber is larger than that of films with EG, which again correlates to the NMR studies in which larger intensities were observed for OH-PD signals than for OH-EG.

**Figure 7:**
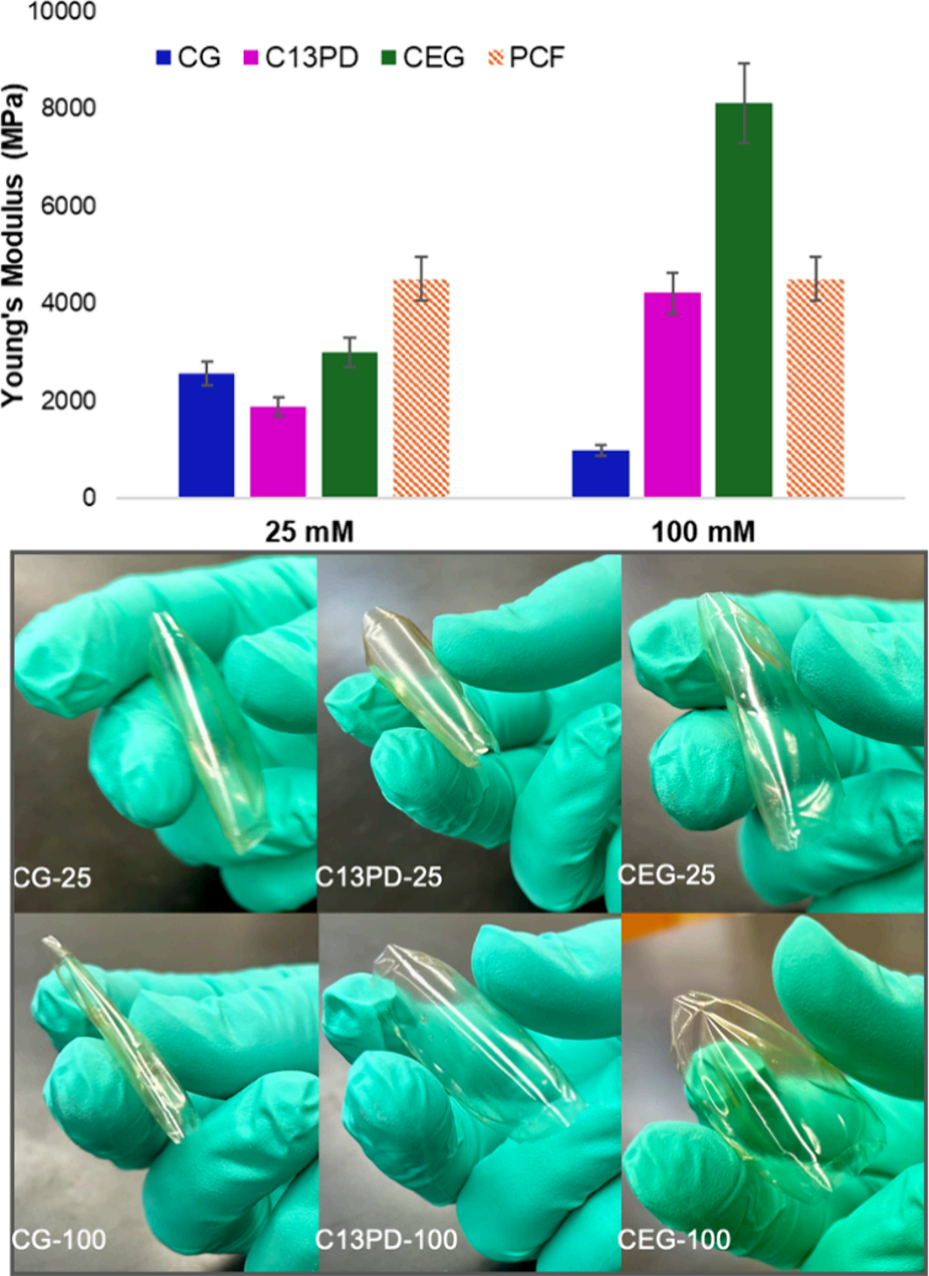
(Top) Young’s moduli of CG (blue), C13PD (pink), and CEG (green) at 25 and 100 mM polyol and PCF (orange) determined from oscillatory stress–strain curves recorded at 1 Hz (Figures S11–S13). (Bottom) Images of film flexibility of 25 mM and 100 mM polyol-containing films.

Insights into the structural interactions at both the molecular and polymer levels have shown that 1,3-PD and EG interact differently with GlcN and chitosan than Glyc. This suggests that there could be different plasticization mechanisms present, depending on the structure of the polyol. In particular, we anticipated that the identity of the polyol would influence the stiffness and fragility of the chitosan films. Thus, dynamic mechanical analysis (DMA) was used to investigate the viscoelastic behavior and stiffness of these films (Figure 7, Figures S12–S14). Previously, we reported a decrease in the Young’s modulus of chitosan in the presence of Glyc,^27^ which was consistent with other reports in the literature.^28,29^ Notably, this property is dependent on the concentration of Glyc. As the concentration of Glyc increases, the film’s resistance to strain decreases, which corresponds to lower Young’s moduli and more flexible materials (Figure S12). With EG and 1,3-PD, we observed the opposite trend. As the concentration of either diol is increased in the film, the flexibility decreases, and the material becomes more stiff and brittle (Figures S13 and S14). Interestingly, we found that films made with 25 mM polyol were more flexible and had relatively similar Young’s moduli (∼2000–2500 MPa), which were all lower than pure chitosan films (PCF = ∼3981 MPa) (Figure 7). When the concentration of polyol is increased, however, we observe a dramatic increase in resistance to strain for C13PD and CEG and a notable decrease for CG. This is reflected in the Young’s moduli for these materials at 100 mM polyol (CG = ∼987 MPa, C13PD = ∼4217 MPa, and CEG = ∼8116 MPa) and in their visual response to an external stress (Figure 7). Overall, these findings indicate that the flexibility of the chitosan films is dependent on the concentration of the polyol, with Glyc films becoming more flexible at higher concentrations and EG or 1,3-PD films becoming more brittle. The chitosan films made with 1,3-PD are more flexible than those with EG, but they are still stiffer than Glyc films.

Comparing these mechanical data to the NMR studies discussed above leads to some interesting conclusions about the presence of different plasticization mechanisms and the resulting material properties. At low concentrations of diol, the NMR studies would suggest that EG and 1,3-PD interact differently with GlcN than Glyc, which results in different mechanisms of plasticizing chitosan. While Glyc promotes strong GlcN–GlcN intermolecular interactions suggesting the gel theory of plasticization,^26^ with EG and 1,3-PD, intramolecular GlcN interactions are retained and intermolecular GlcN–GlcN interactions are not observed. These spectral characteristics are consistent with EG and 1,3-PD plasticizing chitosan via the lubricity theory mechanism. Notably, the differences in the mechanical properties at low concentrations of polyol (25 mM) are minimal, suggesting that all the polyols are plasticizing chitosan and that both plasticization mechanisms are effective. However, at high concentrations of polyol (100 mM), the observed mechanical properties of chitosan films are distinctly different, with EG having a significantly higher Young’s modulus than chitosan alone. On the molecular level, we observe strong interactions between the OHs of EG and the OHs of GlcN at high concentrations, which we interpret as EG surrounding GlcN molecules. This is consistent with the aforementioned observations of the polymeric system. We theorize that the increase in Young’s modulus at higher concentrations of EG is the result of increased interchain polymer–polymer interactions, which EG both cannot disrupt and may promote. These results also indicate that at all concentrations evaluated, Glyc can plasticize chitosan via the gel theory, whereas the plasticization of chitosan with EG and 1,3-PD is concentration-dependent.

**Figure 8:**
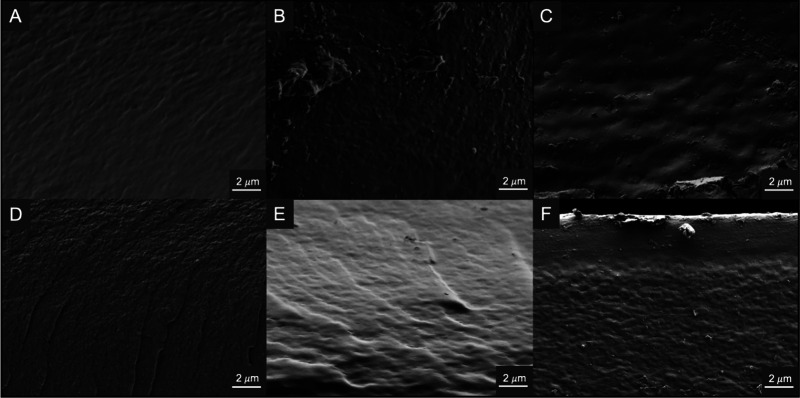
SEM cross-sectional morphologies of 25 and 100 mM Glyc-, 1,3-PD-, and EG-containing chitosan films. (A) CG-25, (B) C13PD-25, (C) CEG-25, (D) CG-100, (E) C13PD-100, and (F) CEG-100.

Surface and cross-sectional morphologies were obtained using scanning electron microscopy (SEM). Surface images of all films show a similar trend with increasing smoothness as the polyol concentration increases when compared to PCF, with minor granularities or surface defects from the drying process (Figures S15 and S16). However, these morphologies do not necessarily give any insight into the differences in physical properties that are observed for the films. Cross-sectional images are much more valuable when correlating the differences in physical properties as a result of the morphologic changes with increased polyol in the films (Figure 8). PCF displays a heterogeneous morphology with visible pore-like structures, where CG-25 shows a more uniform morphology than either C13PD-25 or CEG-25. Interestingly, at high concentrations of polyol, CG-100 exhibits more structure than C13PD-25 or CEG-25 suggesting that different modes of plasticization could be present, which result in different mechanical properties, as was evidenced in the DMA data.

**Figure 9:**
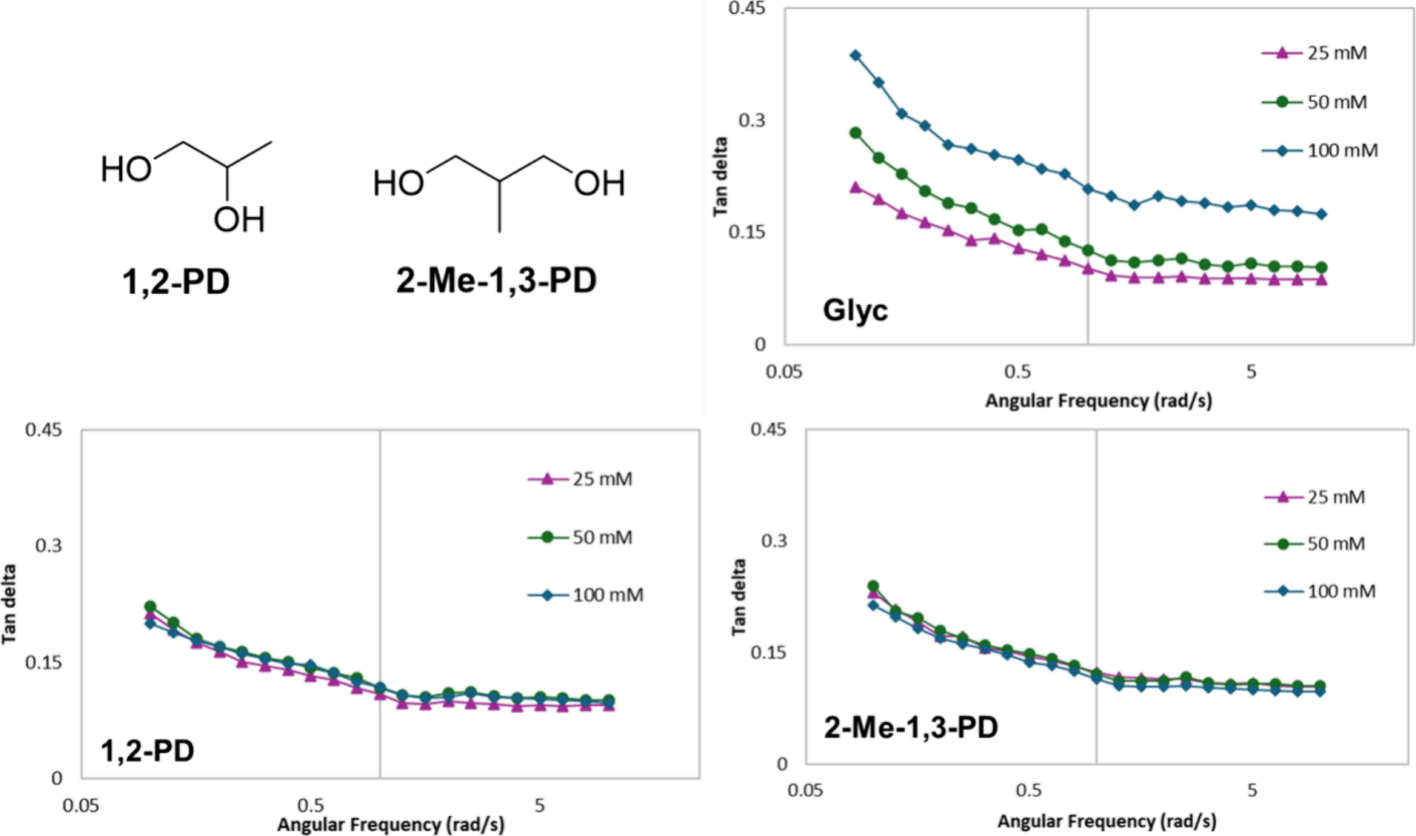
Structures of 1,2-PD and 2-Me-1,3-PD and oscillatory frequency sweeps at 0.5% strain for chitosan films with 25–100 mM Glyc, 1,2-PD, or 2-Me-1,3-PD.

The differences in mechanical and morphological properties of the polymeric system, despite some similarities in intermolecular polyol–GlcN and polyol–chitosan interactions, suggest that secondary interactions may be the key to the plasticization mechanism. In particular, we theorized that the third OH of Glyc is critical for forming secondary hydrogen bonding interactions, while other reports have emphasized the importance of hydrophobicity for effective chitosan plasticization.^21^ However, both 1,3-PD and EG have the capability to form hydrogen bonds and expose a hydrophobic region when bound to the polymer, but they do not show the same plasticization as Glyc. To probe this further, we evaluated the impact of adding a carbon to the diols at the appropriate positions to mimic Glyc. Specifically, we prepared chitosan films containing either 1,2-propanediol (1,2-PD) and 2-methyl-1,3-propanediol (2-Me-1,3-PD) and compared them to those made with Glyc (Figure 9). Films containing 1,2-PD and 2-Me-1,3-PD showed a reduction in the water content of the films when compared to CEG and C13PD films, respectively, when analyzed using thermogravimetric analysis (TGA) (Table S1). Glyc, the most hydrophilic of the polyols investigated, has been shown to improve the ability of the films to retain moisture. As anticipated, we found that Glyc-containing films had increased water content relative to all other films, a finding that has been previously observed.^13^ These results are consistent with the increased hydrophobicity of 1,2-PD and 2-Me-1,3-PD relative to that of the other polyols. DMA experiments were performed on these films, and tan delta values were measured as a function of frequency. As anticipated, the tan delta of films containing Glyc increases as a function of Glyc concentration, doubling in value from 25 to 100 mM (from 0.102 to 0.207 at 1 rad/s). This indicates that the films are becoming less solid-like and more flexible with increasing amounts of Glyc. In contrast, films made with 1,2-PD or 2-Me-1,3-PD show little change in tan delta with increasing concentration, having average values of ∼0.113 and ∼0.116 at 1 rad/s, respectively. These results indicate that increasing hydrophobicity does not result in improved plasticization and that specific secondary interactions are indeed critical for plasticizing chitosan.

## Conclusions

In conclusion, we investigated the primary and secondary interactions of polyols on both the molecular level with GlcN and in a polymeric system with chitosan. We used NOESY techniques to evaluate changes in the molecular-level interactions of 1,3-PD and EG with GlcN and compared them to those of Glyc–GlcN interactions. These studies highlighted the differences in interactions with the NH and OH functionalities of GlcN based on primary diol binding mode suggesting that 1,3-diol binding prefers NH interactions, while 1,2-diol binding prefers OH interactions. The molecular-level data was complemented by an investigation of the polymer systems CG-25-100, C13PD-25-100, and CEG-25–100, which were analyzed using ATR-IR, DMA, SEM, and TGA. At low concentrations of diol, the properties of C13PD-25 and CEG-25 were comparable to CG-25. However, as the concentration of polyol was increased, films containing 1,3-PD or EG did not exhibit the same mechanical or morphological properties as those containing Glyc. These combined results suggest the potential of different plasticization mechanisms within the system. While findings with Glyc continue to fit the gel theory of plasticization, 1,3-PD and EG showed significant differences with regard to GlcN–GlcN aggregation. That is, even at high concentrations of 1,3-PD and EG, no intermolecular GlcN–GlcN interactions were observed, indicating that GlcN molecules are not being aggregated in the presence of the diols. Instead, we theorize that the diols effectively surround individual GlcN molecules, which indicates the potential for lubricity theory in the plasticized systems.

The differences observed with the diols relative to Glyc also emphasized the importance of secondary interactions in plasticization. 1,2-PD and 2-Me-1,3-PD were incorporated into chitosan to probe the impact of adding a methyl substituent to the diols at the appropriate positions to mimic Glyc. These materials were analyzed using DMA and showed no mechanical changes with increasing diol concentration in contrast to Glyc. These combined analyses provide strong evidence that both primary and secondary interactions are important for the effective plasticization of chitosan. That is, changing from hydrogen bonding to hydrophobic secondary interactions may result in a shift in mechanism from gel to lubricity theory. Density-functional theory calculations are currently being employed to further explore these mechanisms in more depth. Understanding plasticizer–biopolymer interactions is an important step for tuning the properties of these biomaterials in future applications.

## Experimental Section

### Materials

Chitosan with a degree of deacetylation DD = 75–85% and a molecular weight range of 50,000–190,000 was used as received from Sigma-Aldrich. d-Glucosamine hydrochloride (GlcN), glycerol (Glyc), 1,3-propanediol (1,3-PD), ethylene glycol (EG), 1,2-propanediol (1,2-PD), 2-methyl-1,3-propanediol (2-Me-1,3-PD), and deuterated NMR solvents were also used as received from Sigma-Aldrich.

### Film Formation

Chitosan films were prepared by diluting equal volumes of 2 wt % stock chitosan solution (20.0 g of chitosan in 980 g of 1% aqueous acetic acid) and 1% aqueous acetic acid. Polyol-containing films were prepared in a similar fashion from 2 wt % stock chitosan solution, 1% aqueous acetic acid, and a stock solution of polyol also in 1% aqueous acetic acid (200.0 mM). The resulting solutions contained 1 wt % chitosan with a final concentration of polyol ranging from 25 to 100 mM. All of the solutions were stirred at 80 °C for 1 h to ensure complete homogeneity of the casting solutions. All films were cast into polystyrene dishes by using 5 mL of the prepared solutions. The films were dried at 60 °C overnight.

### Infrared and Nuclear Magnetic Resonance Spectroscopy

FT-IR spectra of the chitosan films were obtained using a Thermo Scientific Nicolet iS10 spectrometer, equipped with a SMART iTX attenuated total reflection (ATR) accessory. The FTIR spectra were recorded from 400 to 4000 cm^–1^ with 32 scans and a resolution of 4 cm^–1^. Nuclear magnetic resonance (^1^H NMR and NOESY) spectra were obtained using a Bruker Avance DPX-400 NMR spectrometer in *d*_6_-DMSO; chemical shifts are reported in parts per million downfield from tetramethylsilane (δ scale). NOESY measurements were recorded using the NOESYGPPHPP pulse protocol with a mixing time of 300 ms. Data were obtained with a 90° pulse of 10 μs and a relaxation delay of 2 s. A total of 24 scans with a spectral width of 3998 Hz in each dimension were performed. Cross-peaks for NOE interactions and chemical exchange (EXSY) were distinguished by phase (i.e., NOE = negative, EXSY = positive).

### Thermal, Mechanical, and Morphological Analysis

Thermogravimetric analysis (TGA) was performed using a TA Instruments Discovery TGA-550. Samples were heated from 25 to 500 °C with a heating rate of 10 °C/min. The mechanical properties were measured using a TA Instruments DHR-20 rheometer equipped with an ETC and film tension geometry. Axial strain sweeps were performed at 1 Hz and varying strains from 0.01 to 0.1%. Frequency sweeps were performed at 0.05% strain from 0.1 to 100 rad/s. The temperature was maintained at 25 °C. Scanning electron microscopy (SEM) images were obtained using a Zeiss Sigma 300 VP Field Emission Scanning Electron Microscope.

## Supporting Information Available

Additional NOESY cross sections and full spectra, IR spectra, stress–strain curves, SEM images, and TGA data (PDF)

## Abbreviations

GlcN: glucosamine
Glyc: glycerol
1,3-PD: 1,3-propanediol
EG: ethylene glycol
1,2-PD: 1,2-propanediol
2-Me-1,3-PD: 2-methyl-1,3-propanediol
IR: infrared
ATR: attenuated total reflectance
NMR: nuclear magnetic resonance
NOESY: nuclear Overhauser effect spectroscopy
TGA: thermogravimetric analysis
DMA: dynamic mechanical analysis
