# Supplementary material for: Importance of Primary and Secondary Hydrogen Bonding Interactions of Polyols on the Plasticization of Chitosan
Source: ACS Omega. 2024 Sep 24;9(40):41687–95. doi: 10.1021/acsomega.4c05696 (PMC11465282; doi:10.1021/acsomega.4c05696)
Supplement: Supplementary file 1 — ao4c05696_si_001.pdf [file ao4c05696_si_001.pdf]

Supporting Information

The Importance of Primary and Secondary Hydrogen Bonding Interactions of Polyols on the Plasticization of Chitosan

Seth W. Mars, Brandy L. Davidson, Kayla H. Moore, Brycelyn M. Boardman,[‡*] and Gretchen M. Peters[‡*]

Department of Chemistry and Biochemistry, James Madison University, Harrisonburg, Virginia 22807, United States

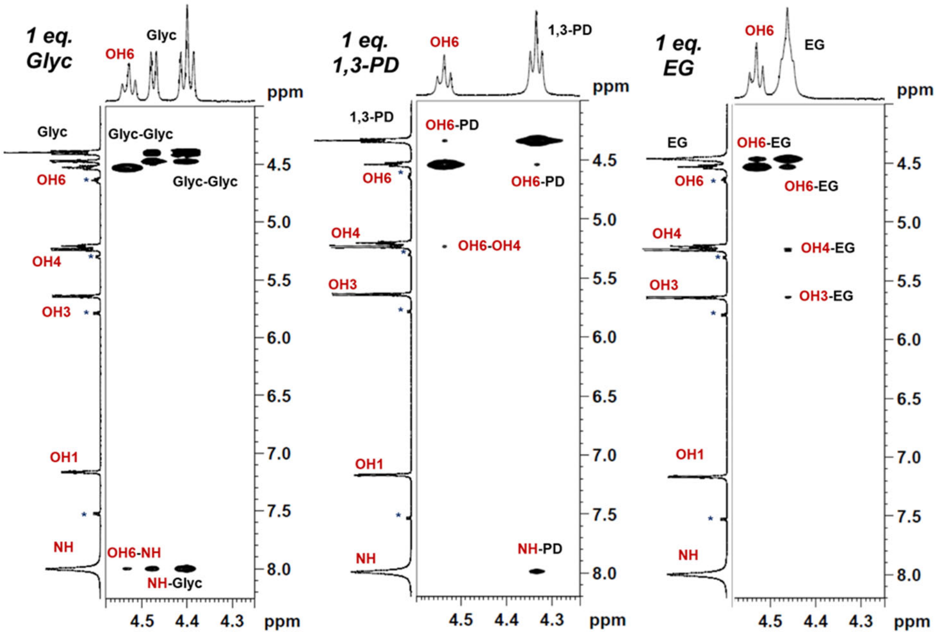

Figure S1. Representative cross sections of the 2D NOESY spectra of the polyol OH's at 12.5 mM GlcN and 12.5 mM Glyc (left), 12.5 mM 1,3-PD (middle), or 12.5 mM EG (right) recorded at 400 MHz (9.4 T). Small peaks for the alpha anomer of GlcN are observed in the spectra, and the corresponding peaks are denoted with an asterisk (*).

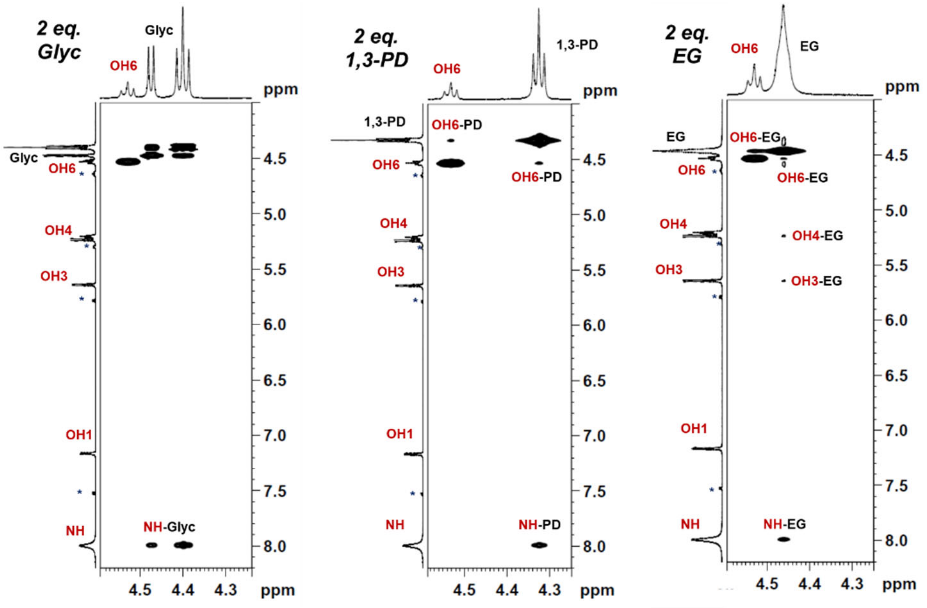

Figure S2. Representative cross sections of the 2D NOESY spectra of the polyol OH's at 12.5 mM GlcN and 25 mM Glyc (left), 25 mM 1,3-PD (middle), or 25 mM EG (right) recorded at 400 MHz (9.4 T). Small peaks for the alpha anomer of GlcN are observed in the spectra, and the corresponding peaks are denoted with an asterisk (*).

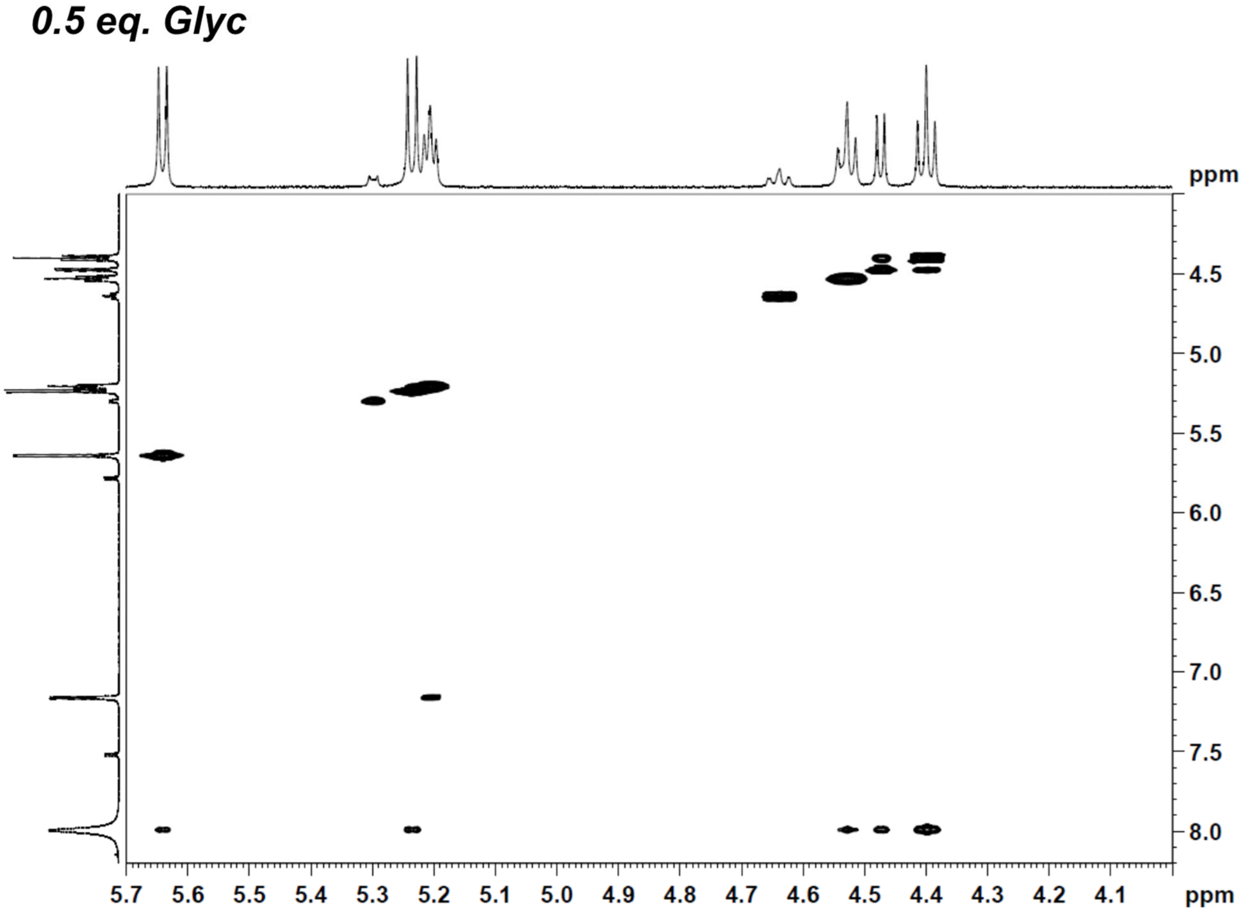

Figure S3. Full 2D NOESY spectra of 12.5 mM GlcN and 6.25 mM Glyc in d6-DMSO recorded at 400 MHz (9.4 T). Full 1D NMR spectrum can be found in Figure S17.

Figure S3a. We have previously reported the full 2D NOESY spectra of 12.5 mM GlcN and 12.5 mM Glyc in d6-DMSO recorded at 400 MHz (9.4 T) (*Smith, D.R, et. al. ACS Omega* **2021**, *6*, 39, 25227–25234).

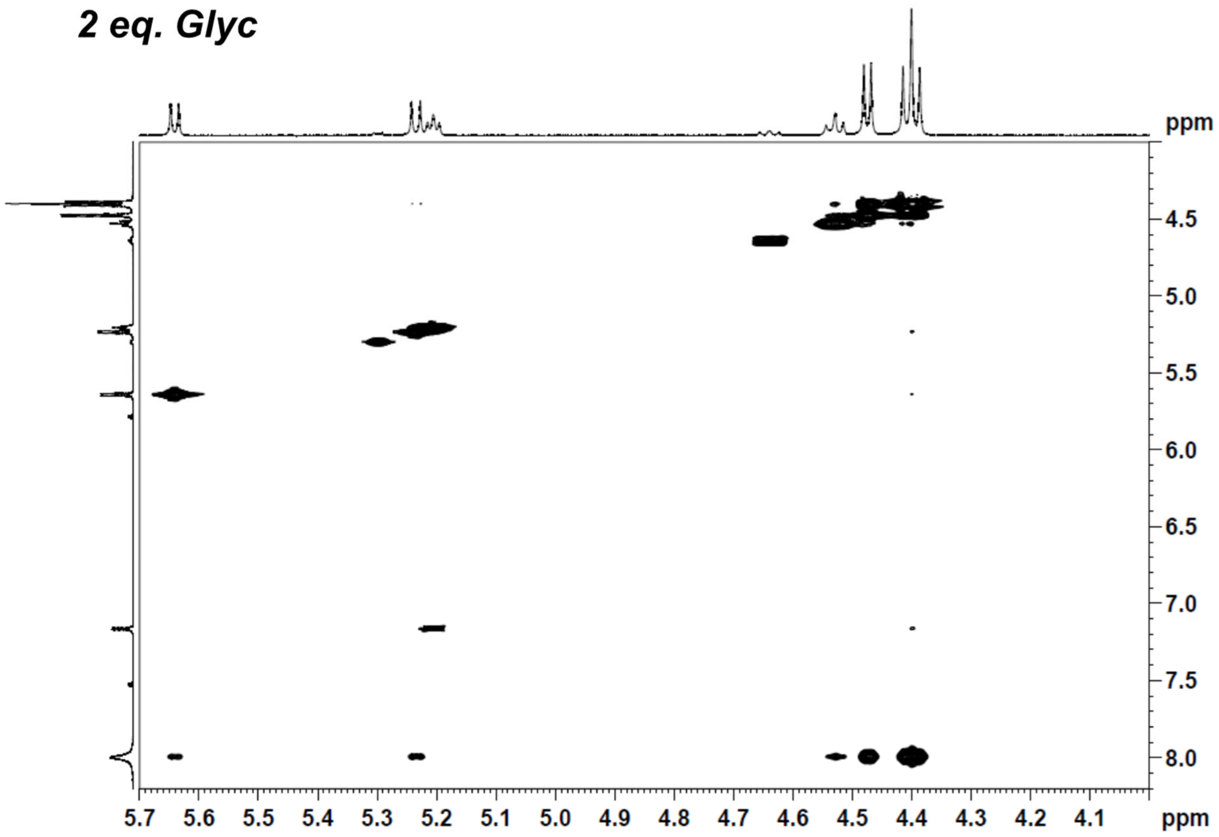

Figure S4. Full 2D NOESY spectra of 12.5 mM GlcN and 25 mM Glyc in d6-DMSO recorded at 400 MHz (9.4 T). Full 1D NMR spectrum can be found in Figure S18.

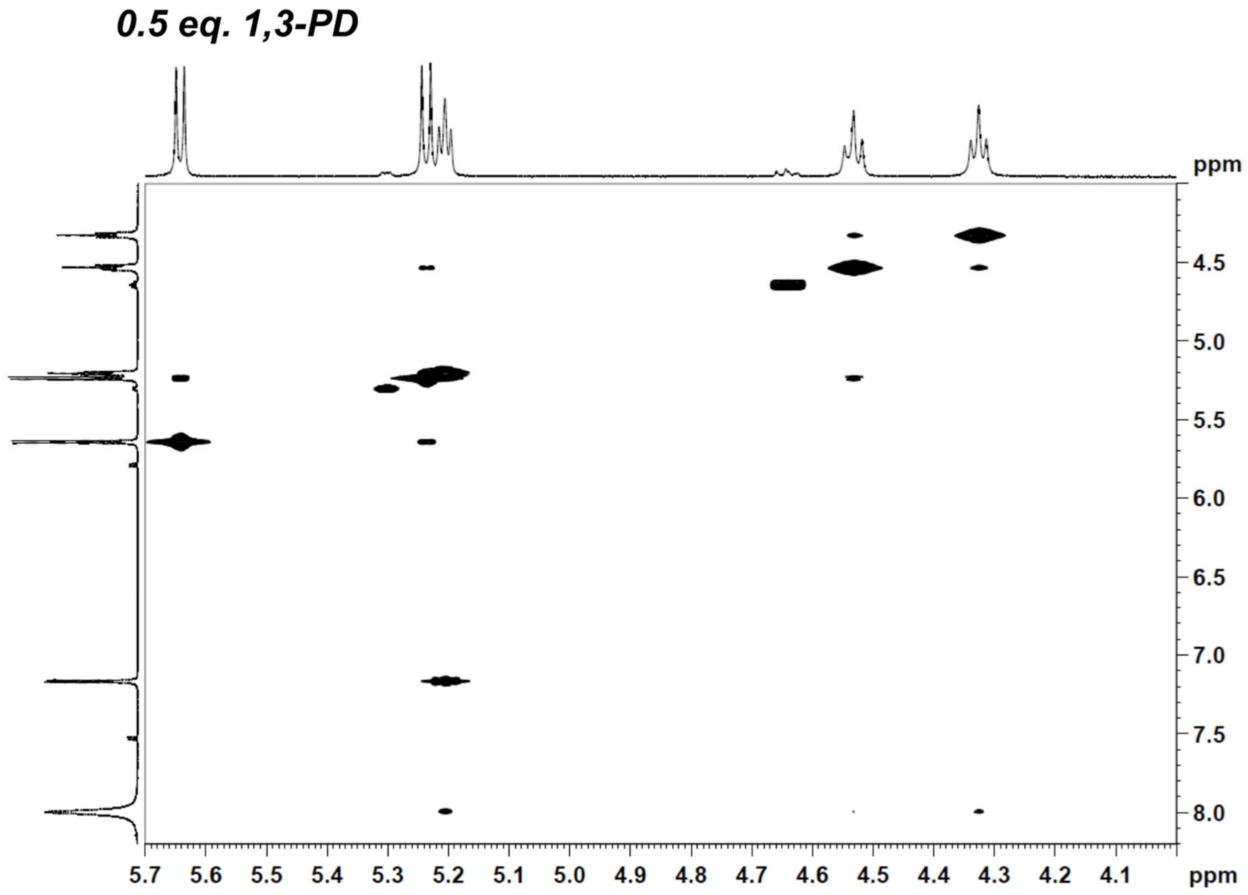

Figure S5. Full 2D NOESY spectra of 12.5 mM GlcN and 6.25 mM 1,3-PD in d6-DMSO recorded at 400 MHz (9.4 T). Full 1D NMR spectrum can be found in Figure S19.

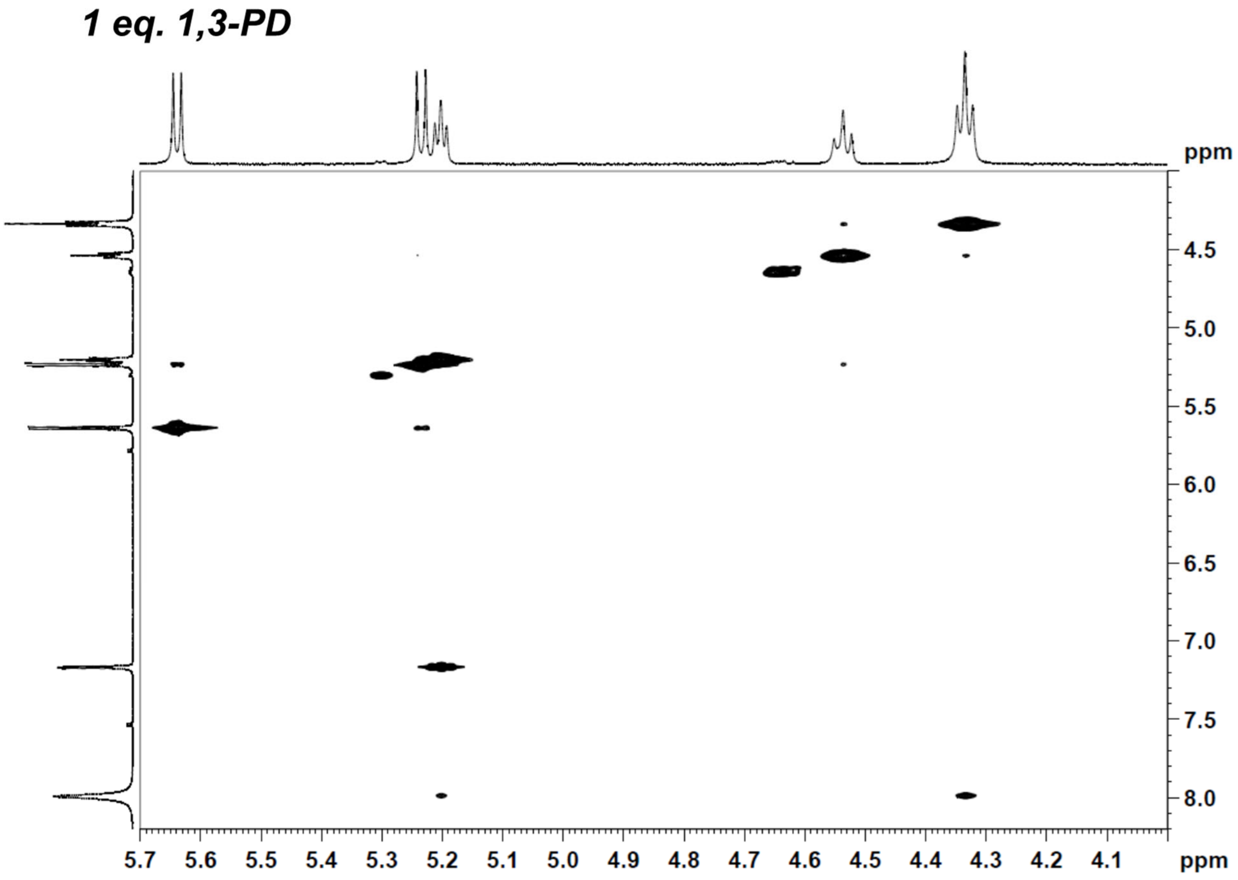

Figure S6. Full 2D NOESY spectra of 12.5 mM GlcN and 12.5 mM 1,3-PD in d6-DMSO recorded at 400 MHz (9.4 T). Full 1D NMR spectrum can be found in Figure S20.

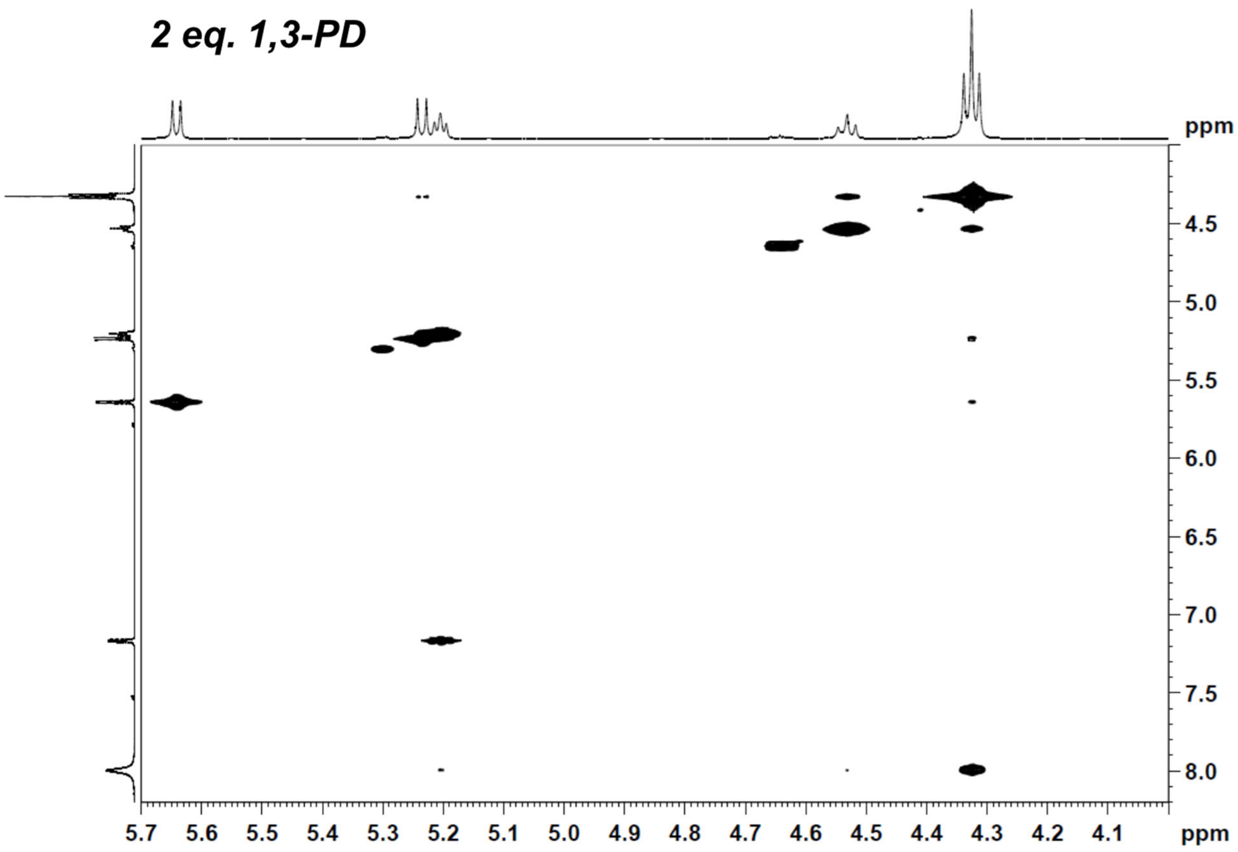

Figure S7. Full 2D NOESY spectra of 12.5 mM GlcN and 25 mM 1,3-PD in d6-DMSO recorded at 400 MHz (9.4 T). Full 1D NMR spectrum can be found in Figure S21.

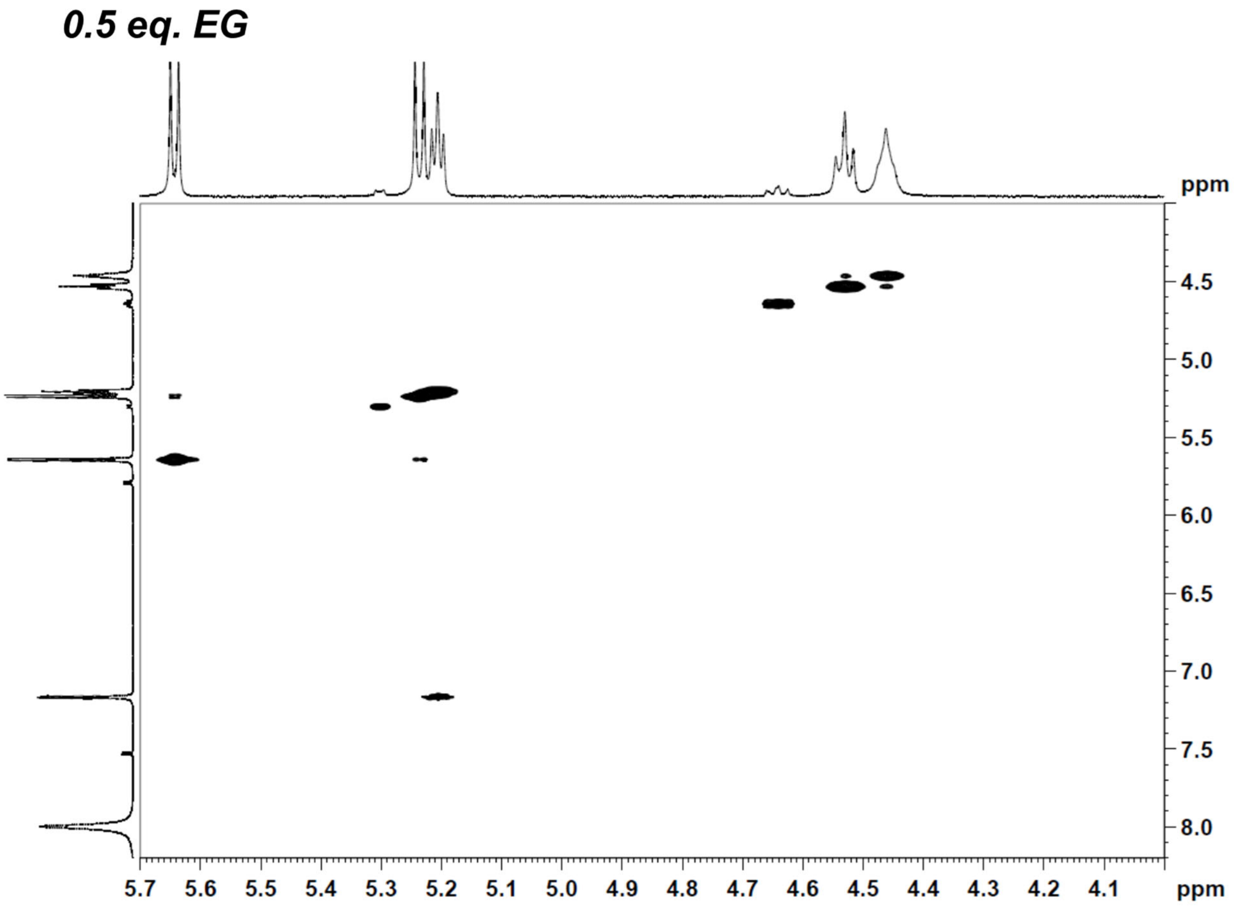

Figure S8. Full 2D NOESY spectra of 12.5 mM GlcN and 6.25 mM EG in d6-DMSO recorded at 400 MHz (9.4 T). Full 1D NMR spectrum can be found in Figure S22.

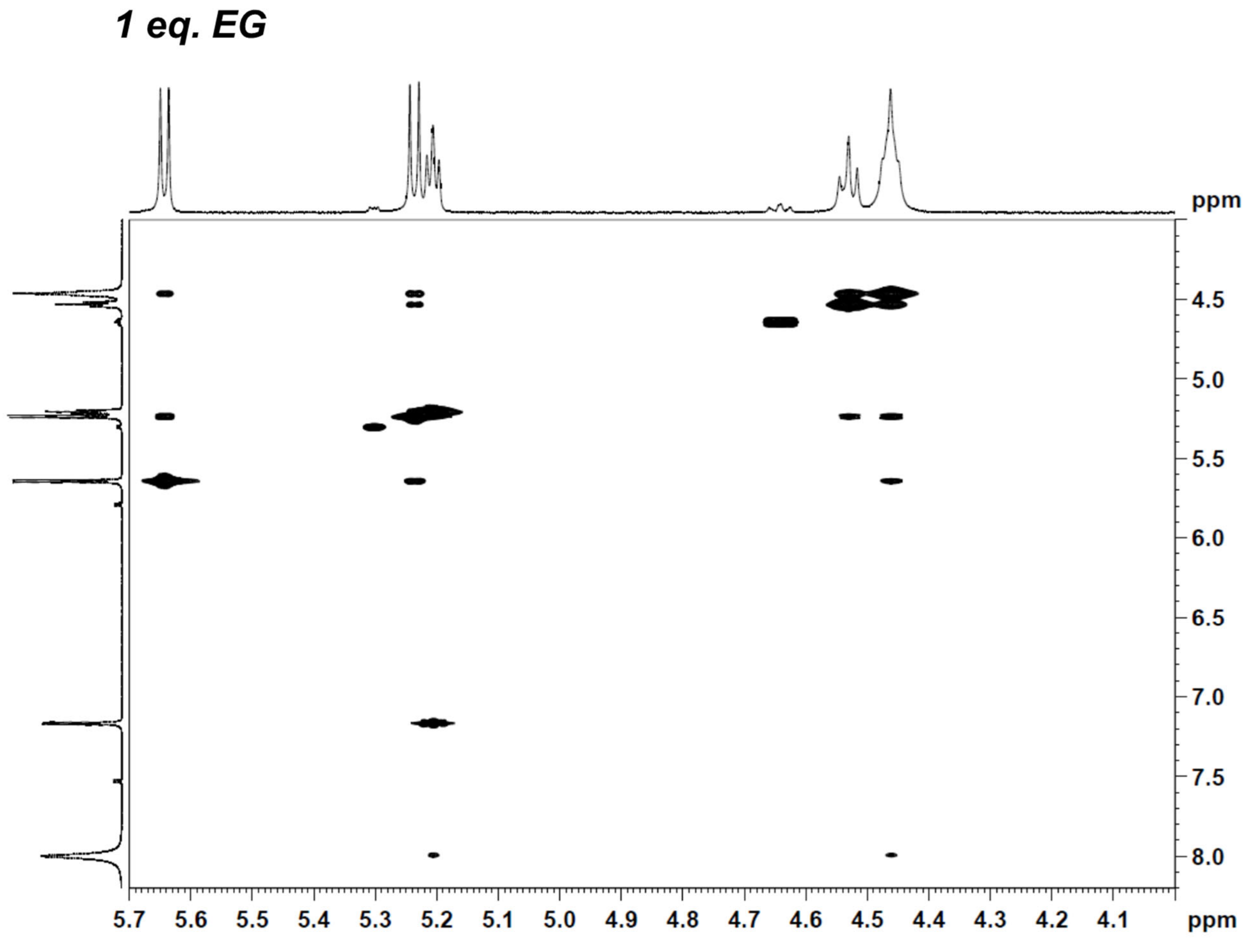

Figure S9. Full 2D NOESY spectra of 12.5 mM GlcN and 12.5 mM EG in d6-DMSO recorded at 400 MHz (9.4 T). Full 1D NMR spectrum can be found in Figure S23.

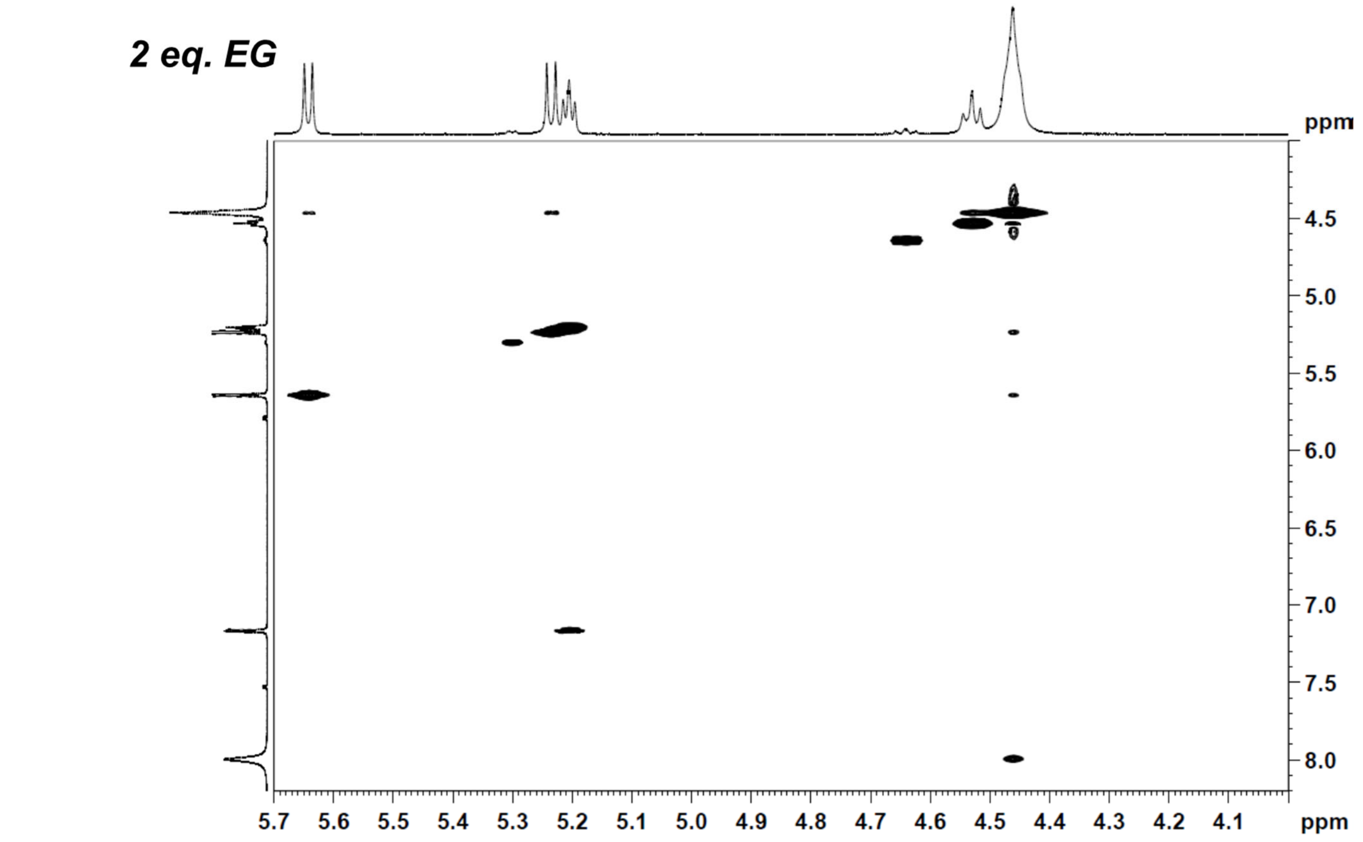

Figure S10. Full 2D NOESY spectra of 12.5 mM GlcN and 25 mM EG in d6-DMSO recorded at 400 MHz (9.4 T). Full 1D NMR spectrum can be found in Figure S24.

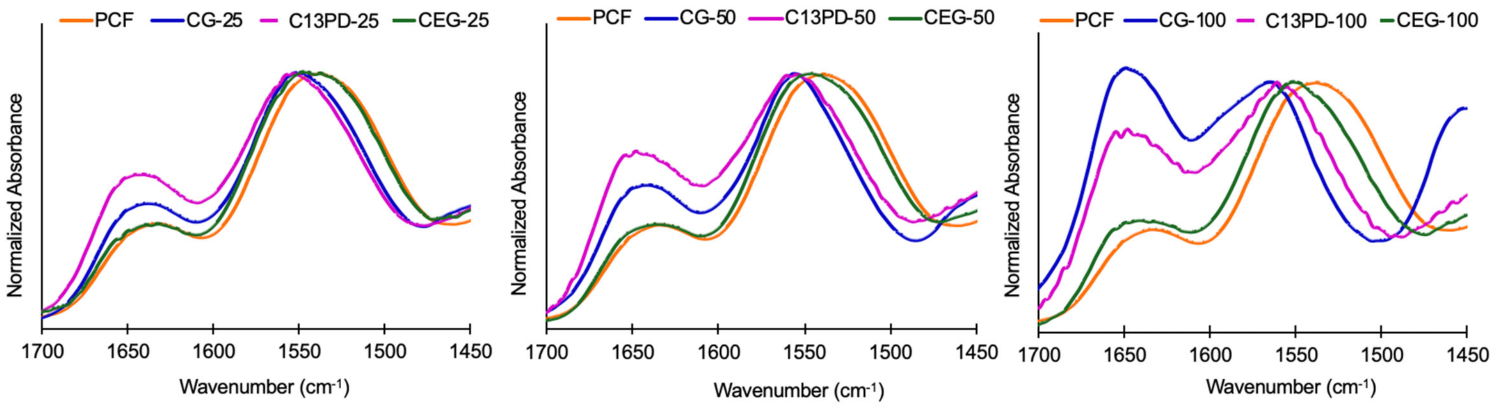

Figure S11. ATR-IR spectral overlay of CN/NH and CO-H combination band region from 1700 cm-1-1450 cm-1 PCF (orange), CG (blue), C13PD (fuchsia), CEG (green) with increasing polyol concentration from 25 mM to 100 mM.

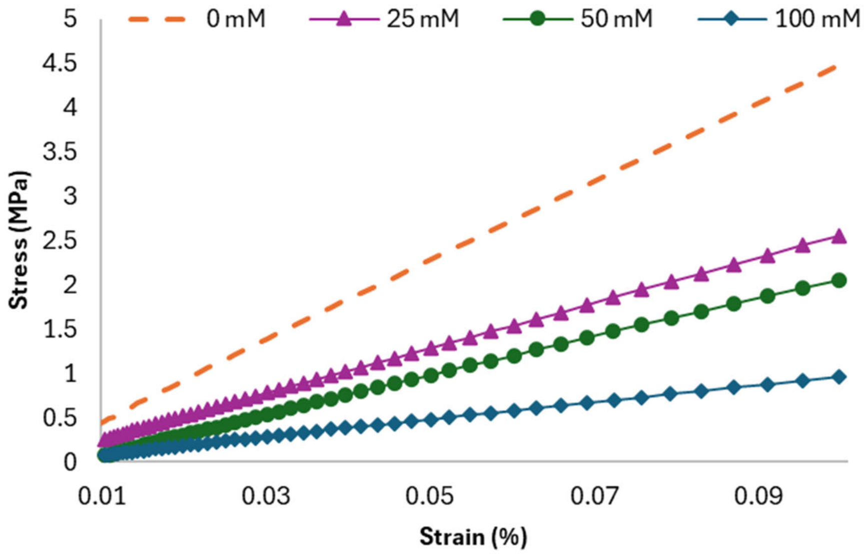

Figure S12. Stress-strain curves of 1% chitosan films with 0 mM (orange), 25 mM (pink), 50 mM (green) and 100 mM (blue) Glyc at 25 °C and 1 Hz.

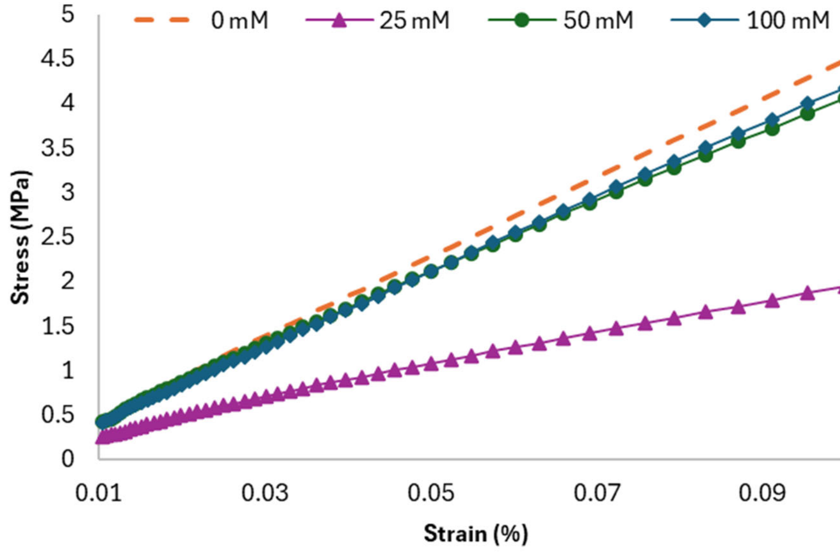

Figure S13. Stress-strain curves of 1% chitosan films with 0 mM (orange), 25 mM (pink), 50 mM (green) and 100 mM (blue) 1,3-PD at 25 °C and 1 Hz.

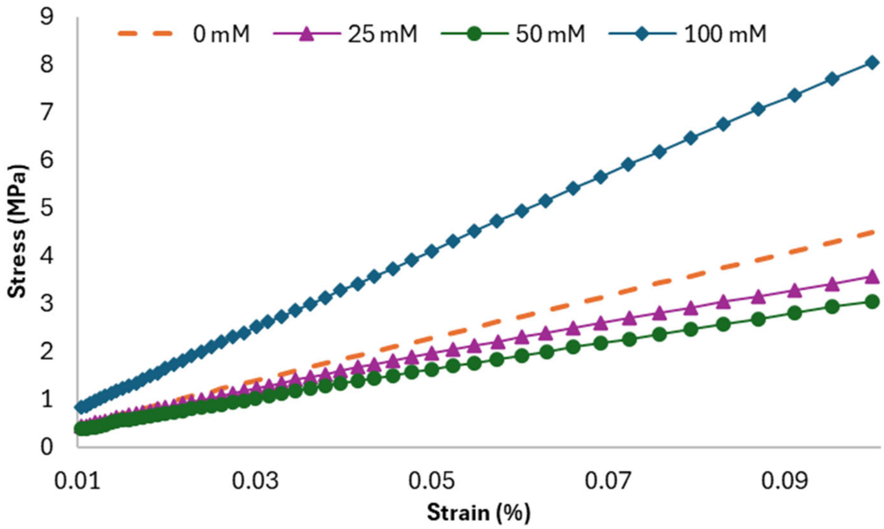

Figure S14. Stress-strain curves of 1% chitosan films with 0 mM (orange), 25 mM (pink), 50 mM (green) and 100 mM (blue) EG at 25 °C and 1 Hz.

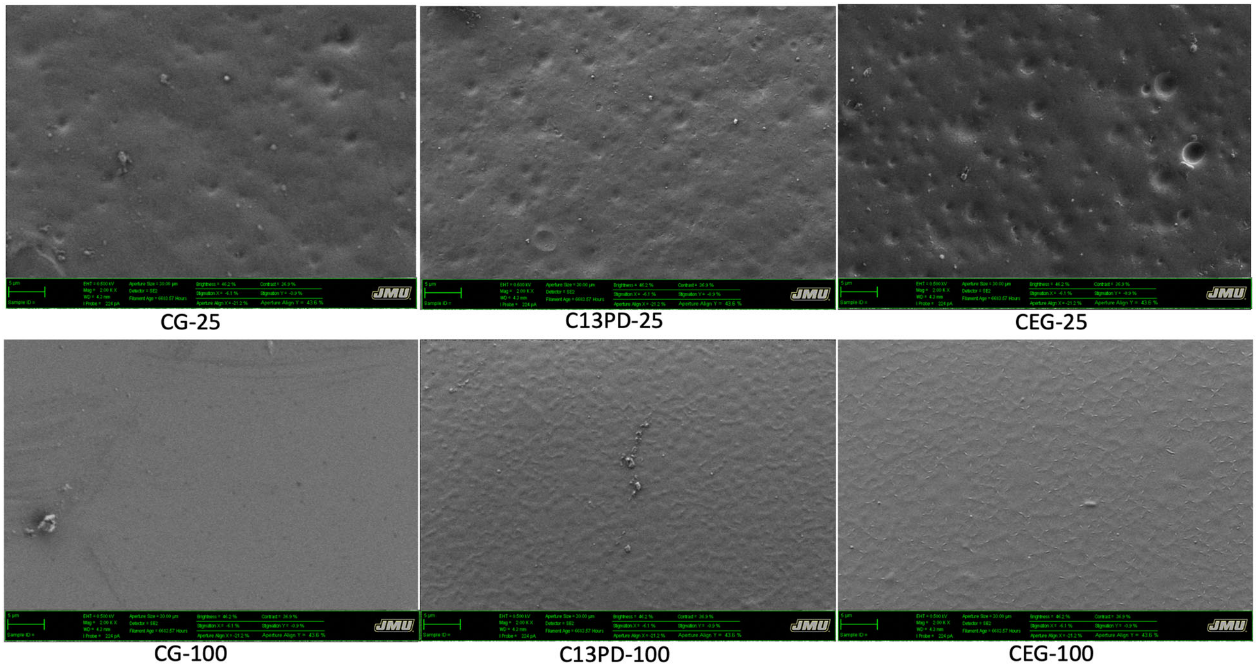

Figure S15. SEM surface morphologies of 1% chitosan films at 25 and 100 mM Glyc (CG-25 and CG-100), 1,3-PD (C13PD-25 and C13PD-100) and EG (CEG-25 and CEG-100).

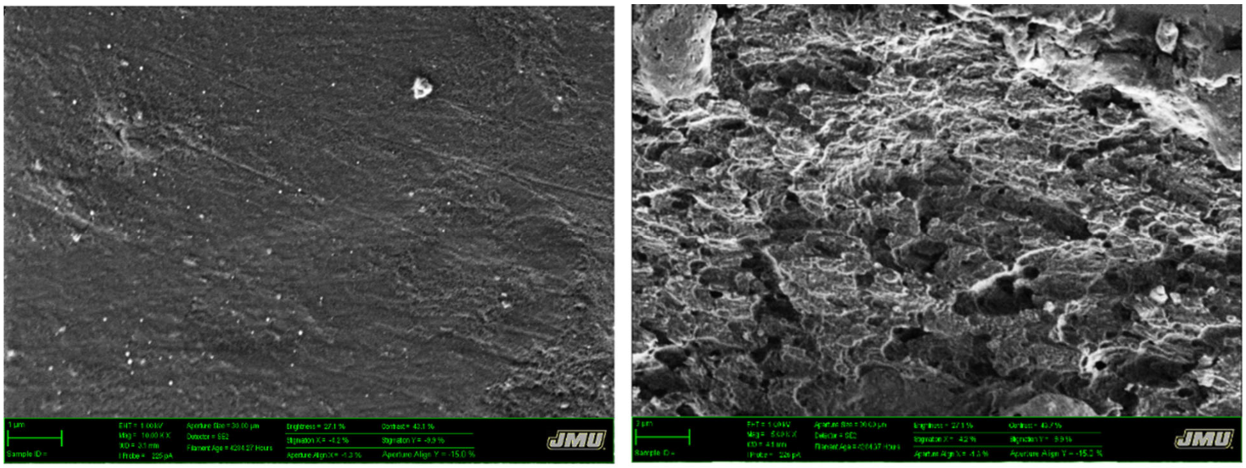

Figure S16. SEM surface (left) and cross-sectional (right) morphologies of 1% chitosan films.

Film	T_{H20} (°C)	WL (%)	T_{max} (°C)	ML (%)
CG-25	21.9	10.7	246.5	48.2
CG-50	21.9	10.9	246.1	40.0
CG-100	23.1	11.2	255.6	42.8
C13PD-25	20.3	10.3	243.9	47.4
C13PD-50	21.6	9.9	247.9	46.8
C13PD-100	21.9	9.7	250.2	46.7
C2M13PD-25	20.3	10.4	244.1	49.8
C2M13PD -50	20.3	9.6	241.9	49.6
C2M13PD-100	24.1	9.3	245.5	51.2
CEG-25	34.4	8.2	245.6	48.4
CEG-50	37.6	8.5	247.5	47.6
CEG-100	37.7	8.4	248.0	47.3
C12PD-25	34.7	7.4	242.4	53.7
C12PD-50	20.7	8.0	239.9	53.2
C12PD-100	35.2	7.7	245.7	50.9

Table S1: TGA of the films continuing different polyols at 25 mM, 50 mM and 100 mM. Values for water loss temperature and mass percent loss (T_{H20}, °C and WL, %) and maximum mass loss temperature and mass percent loss (T_{max}, °C , ML, %) are given.

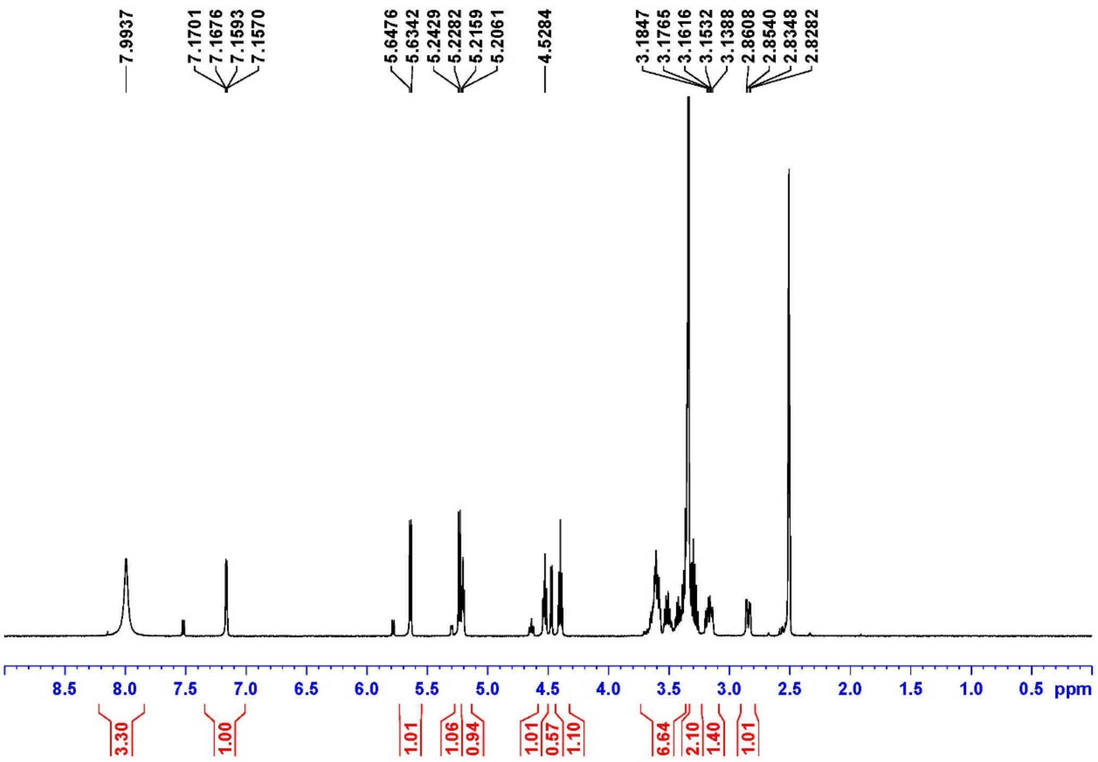

Figure S17. Full 1D NMR spectra of 12.5 mM GlcN and 6.25 mM Glyc in d6-DMSO recorded at 400 MHz (9.4 T).

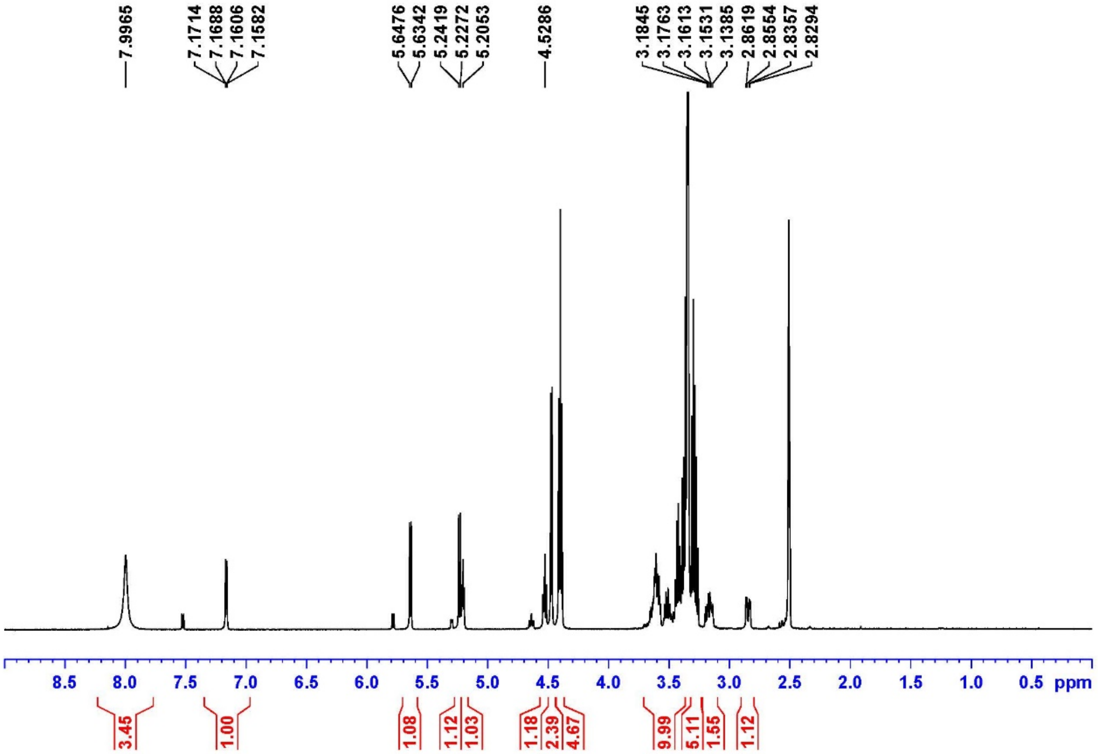

Figure S18. Full 1D NMR spectra of 12.5 mM GlcN and 25 mM Glyc in d6-DMSO recorded at 400 MHz (9.4 T).

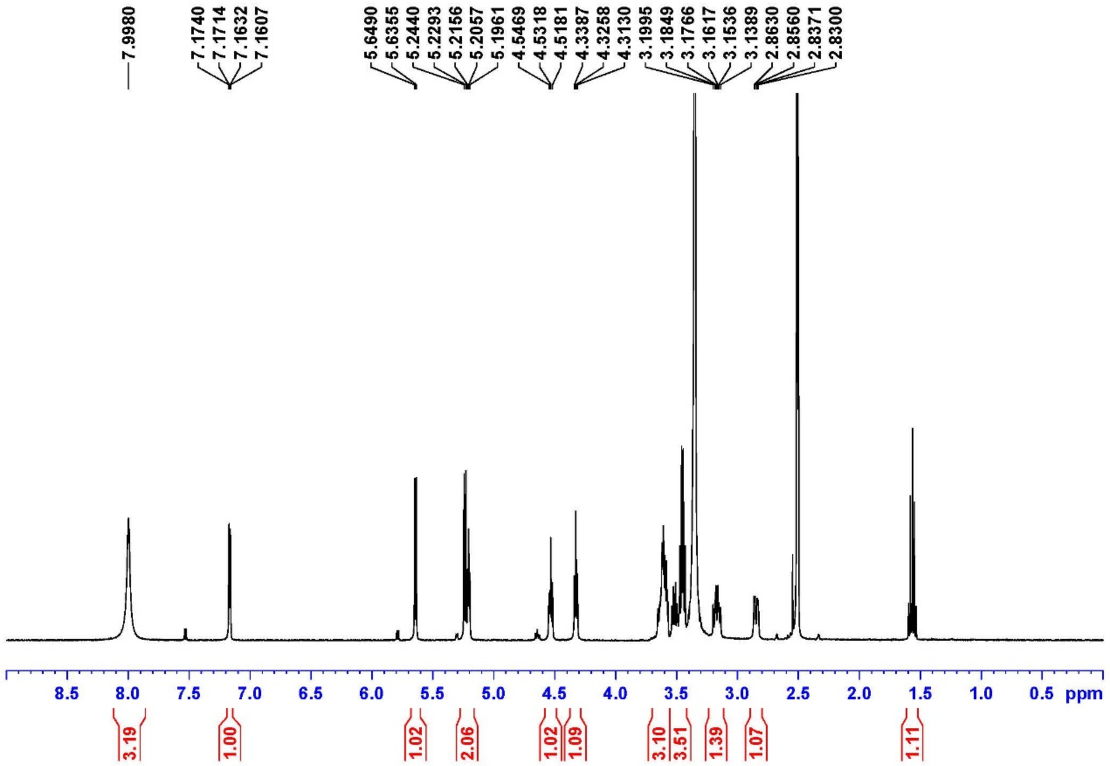

Figure S19. Full 1D NMR spectra of 12.5 mM GlcN and 6.25 mM 1,3-PD in d6-DMSO recorded at 400 MHz (9.4 T).

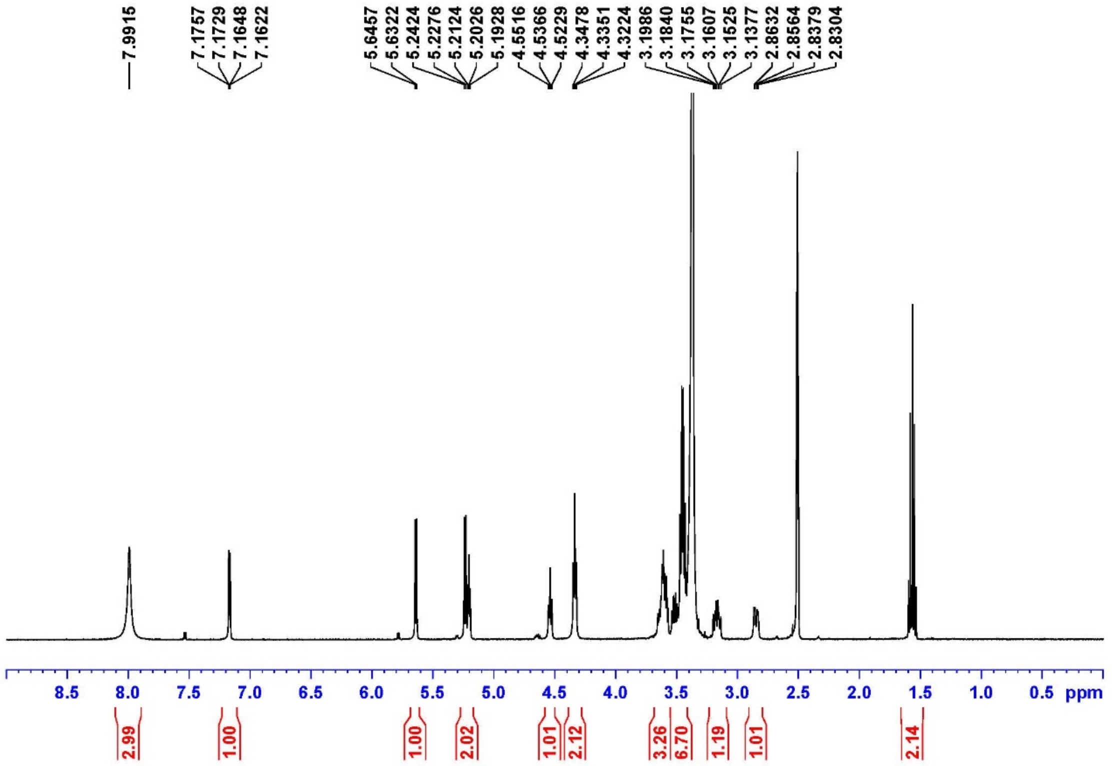

Figure S20. Full 1D NMR spectra of 12.5 mM GlcN and 12.5 mM 1,3-PD in d6-DMSO recorded at 400 MHz (9.4 T).

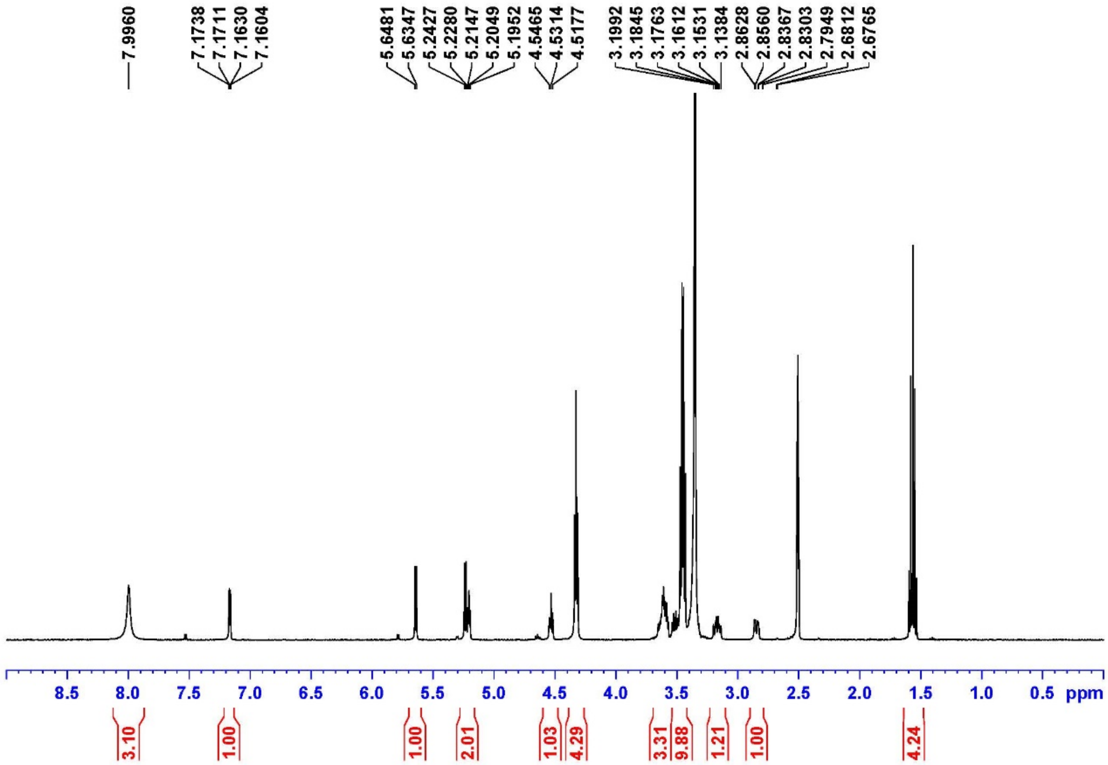

Figure S21. Full 1D NMR spectra of 12.5 mM GlcN and 25 mM 1,3-PD in d6-DMSO recorded at 400 MHz (9.4 T).

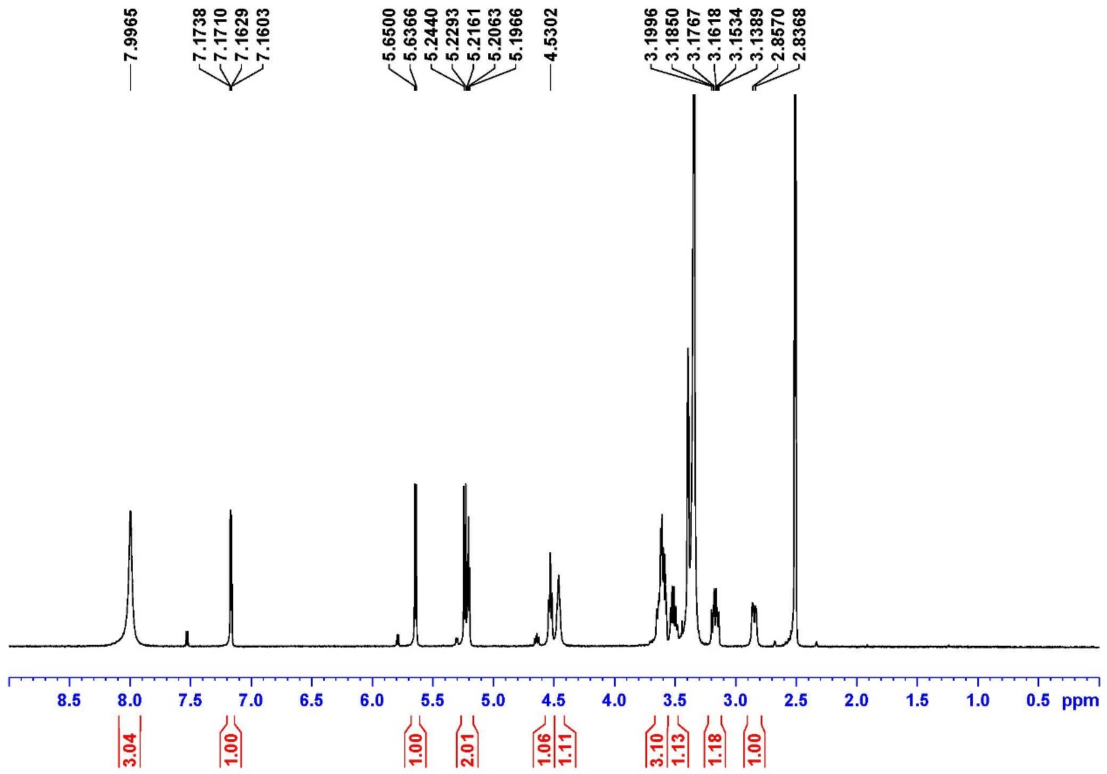

Figure S22. Full 1D NMR spectra of 12.5 mM GlcN and 6.25 mM EG in d6-DMSO recorded at 400 MHz (9.4 T).

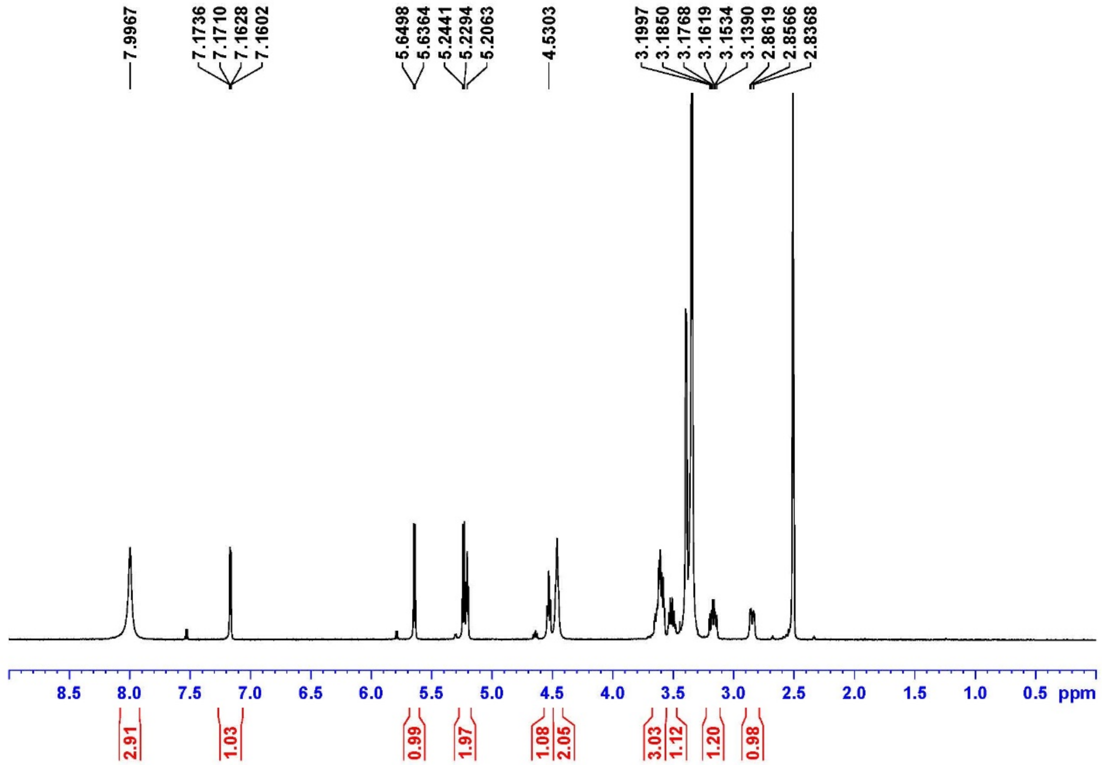

Figure S23. Full 1D NMR spectra of 12.5 mM GlcN and 12.5 mM EG in d6-DMSO recorded at 400 MHz (9.4 T).

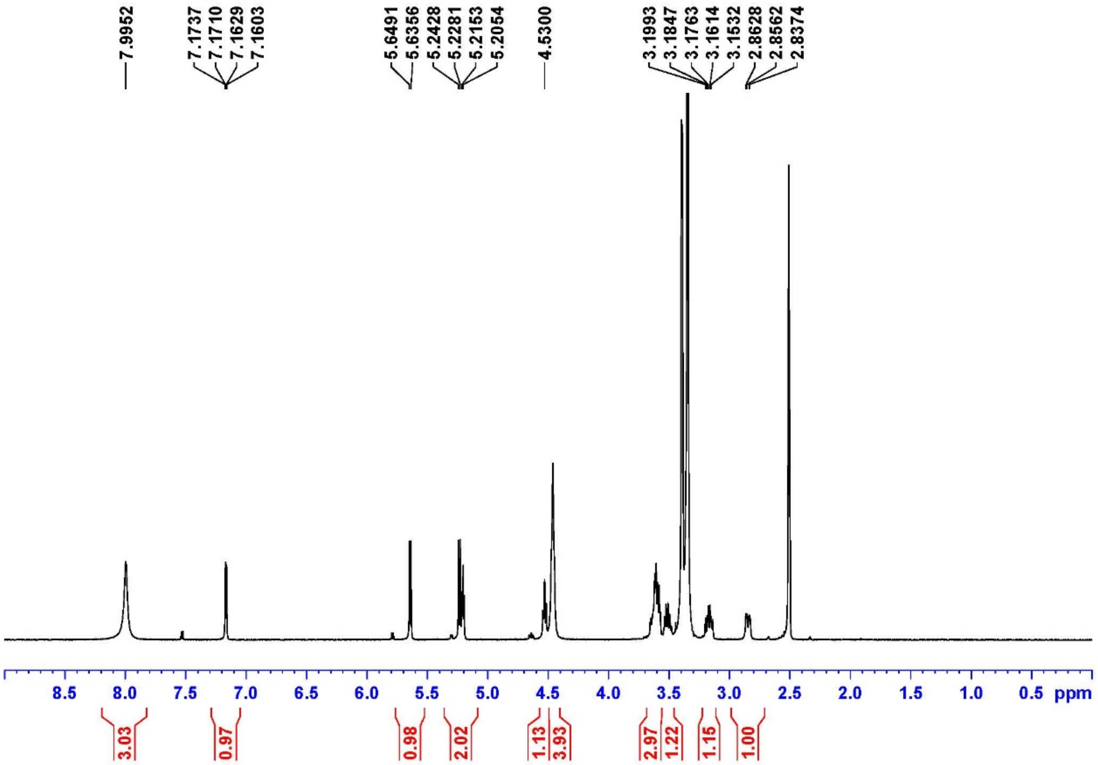

Figure S24. Full 1D NMR spectra of 12.5 mM GlcN and 25 mM EG in d6-DMSO recorded at 400 MHz (9.4 T).
